# Supplementary material for: CLR01 protects dopaminergic neurons in vitro and in mouse models of Parkinson’s disease
Source: Nat Commun. 2020 Sep 28;11:4885. doi: 10.1038/s41467-020-18689-x (PMC7522721; doi:10.1038/s41467-020-18689-x)
Supplement: Supplementary file 1 — Supplementary information [file 41467_2020_18689_MOESM1_ESM.pdf]

Supplementary Information for:

CLR01 protects dopaminergic neurons in vitro and in vivo in human neurons and mouse models of Parkinson's

Bengoa-Vergniory et al. 2020

Supplementary Figures

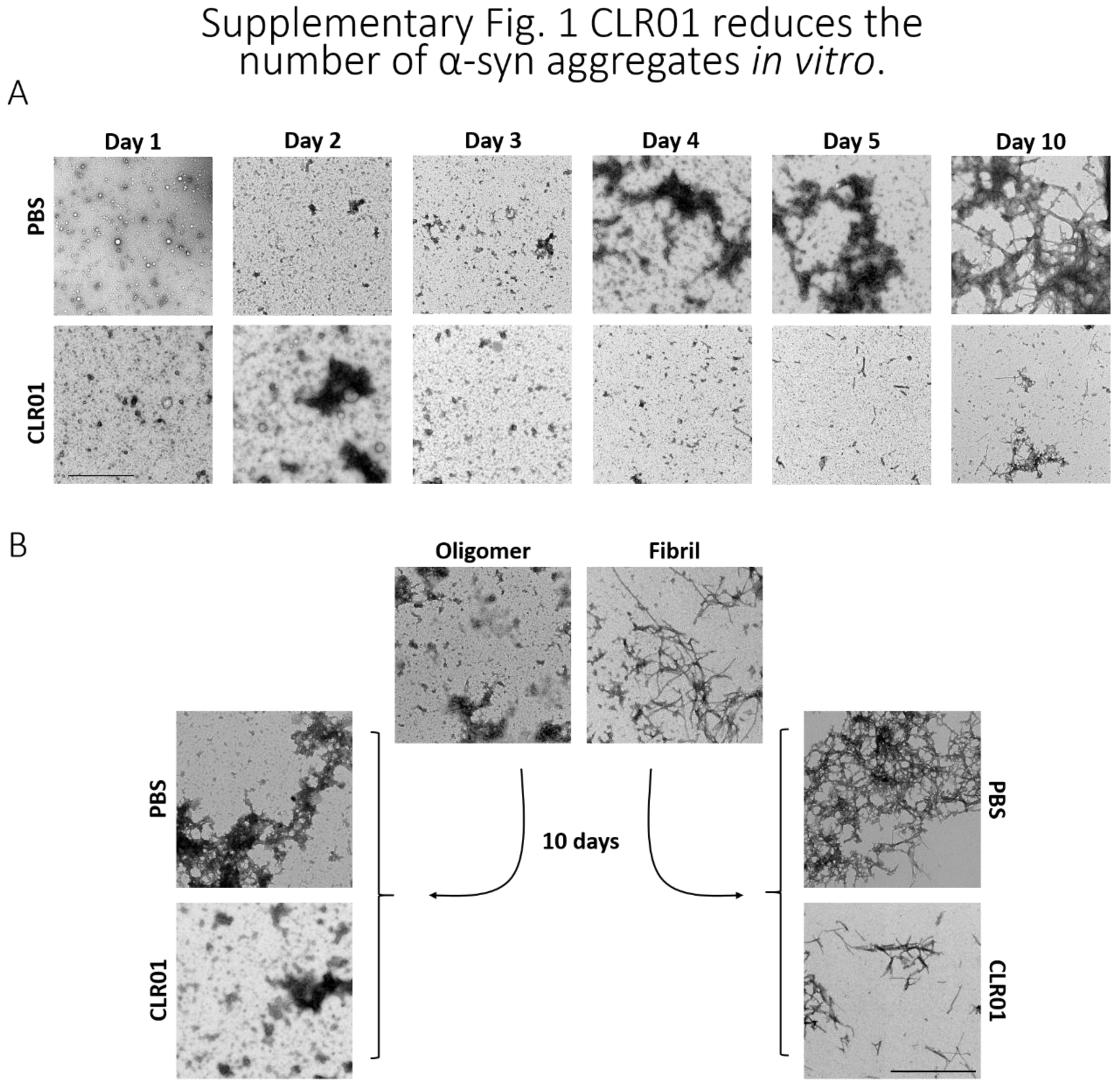

Supplementary Fig. 1. CLR01 reduces the number of α-syn aggregates *in vitro*. **(A)** Representative EM images of α-syn aggregates produced by shaking preparations for 1/2/3/4/5/10 days in the absence/presence of CLR01. **(B)** Representative EM images of α-syn aggregates produced by shaking pre-aggregated oligomers (left) or fibrils (right) for 10 days in the absence/presence of CLR01. **(A and B)** n = 3 independent experiments. Scale bars = 1 µm.

Supplementary Fig. 2 CLR01 reduces the number of α-syn aggregates in Lewy PD brain lysates.

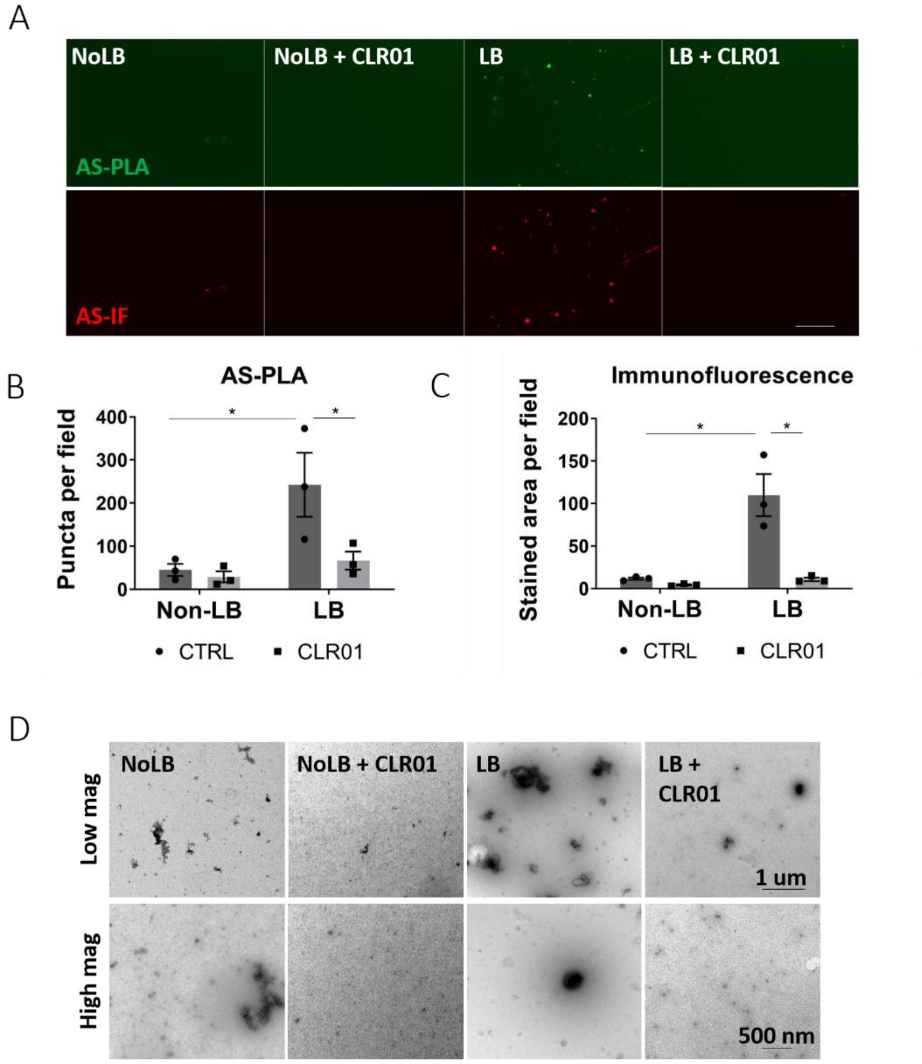

Supplementary Fig. 2. CLR01 reduces the number of α-syn aggregates in PD brain lysates. **(A)** Representative immunofluorescent images of extracts produced by shaking noLB and LB preparations in the absence/presence of CLR01 for 10 days. Top panels (green) represent AS-PLA, and bottom panels (red) represent α-syn immunofluorescence. Scale bars = 50 µm. **(B and C)** The graphs are quantifications of both signals analyzed using a Two-way ANOVA (Sidak), n = 3 independent experiments. * $p < 0.05$. **(D)** Representative EM images of α-syn aggregates produced by shaking noLB and LB preparations in the absence/presence of CLR01 for 10 days (n = 3 independent experiments). Scale bars = 1 µm and 500 nm respectively. IF: immunofluorescence, mag: magnification. B: $F (1, 8) = 5.814$, $p = 0.0159$ and $F (1, 8) = 8.736$, $p = 0.0282$. C: $F (1, 8) = 18.27$ and $F (1, 8) = 17.66$, $p = 0.0011$, $p = 0.0010$. For all appropriate panels data are presented as mean values +/- SEM.

Supplementary Fig. 3 CLR01 reduces oligomeric α-syn *in vitro*.

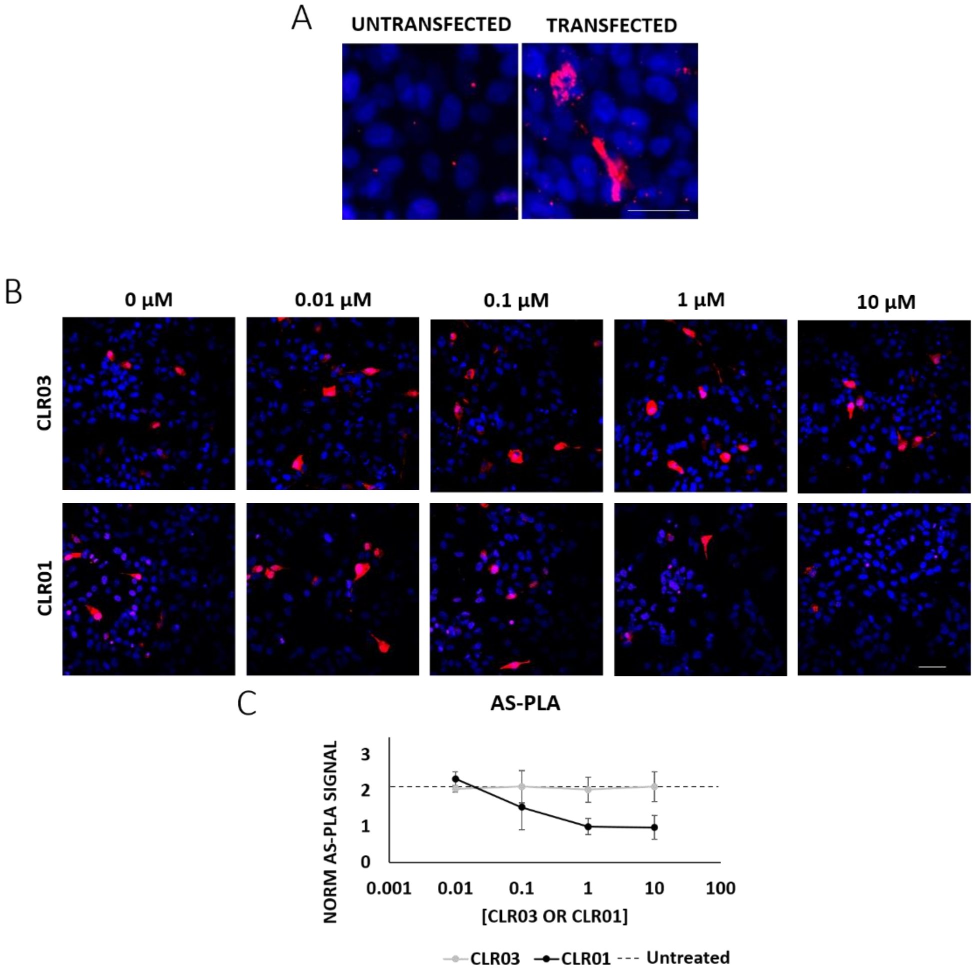

Supplementary Fig. 3. CLR01 reduces oligomeric α-syn *in vitro*. **(A)** Representative immunofluorescence images of AS-PLA (red) in SH-S5Y5 cells either untransfected or transfected with an α-syn expressing plasmid. **(B and C)** Representative immunofluorescence images and quantification of AS-PLA in SH-S5Y5 transfected with an α-syn expressing plasmid. Cells were treated with increasing concentrations of CLR03 or CLR01 in order to determine whether these compounds could dissociate α-syn oligomers in vitro. Best fit for [inhibitor] versus response (variable slope four parameters), IC50 0.08542 μM. n = 3 independent experiments. Scale bars = 25 and 50 μm respectively. For all appropriate panels data are presented as mean values +/- SEM. Norm: normalized.

Supplementary Fig. 4 noLB extracts have minimal toxicity in iPSC-derived dopaminergic cultures.

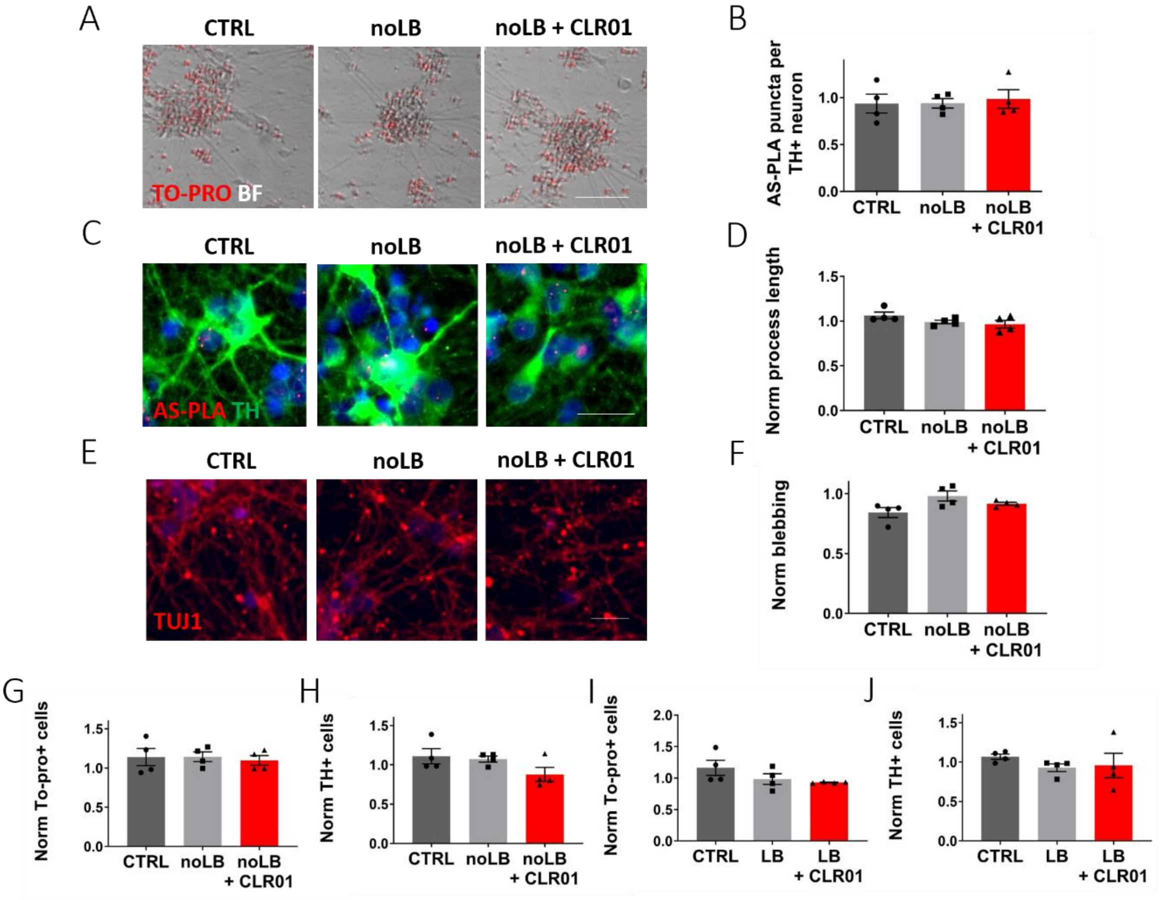

Supplementary Fig. 4. noLB extracts have minimal toxicity in iPSC-derived dopaminergic cultures. **(A, C, E)** Representative brightfield and immunofluorescence images of live and dead cells (labeled by To-pro), dopaminergic cells (TH-positive), AS-PLA (red puncta) and neuronal processes of 4 independent control cell lines analyzed. Cells were treated with noLB extracts, which had been pre-treated with CLR01 or PBS as a negative control. Scale bars = 100, 25, 25 µm respectively. **(B, D, F, G-J)** Quantification of indicated parameters of 4 independent control cell lines analyzed. For all appropriate panels data are presented as mean values +/- SEM. BF: brightfield.

Supplementary Fig. 5 CLR01 reduces oligomeric α-syn in LB extract-treated iPSC dopaminergic cultures.

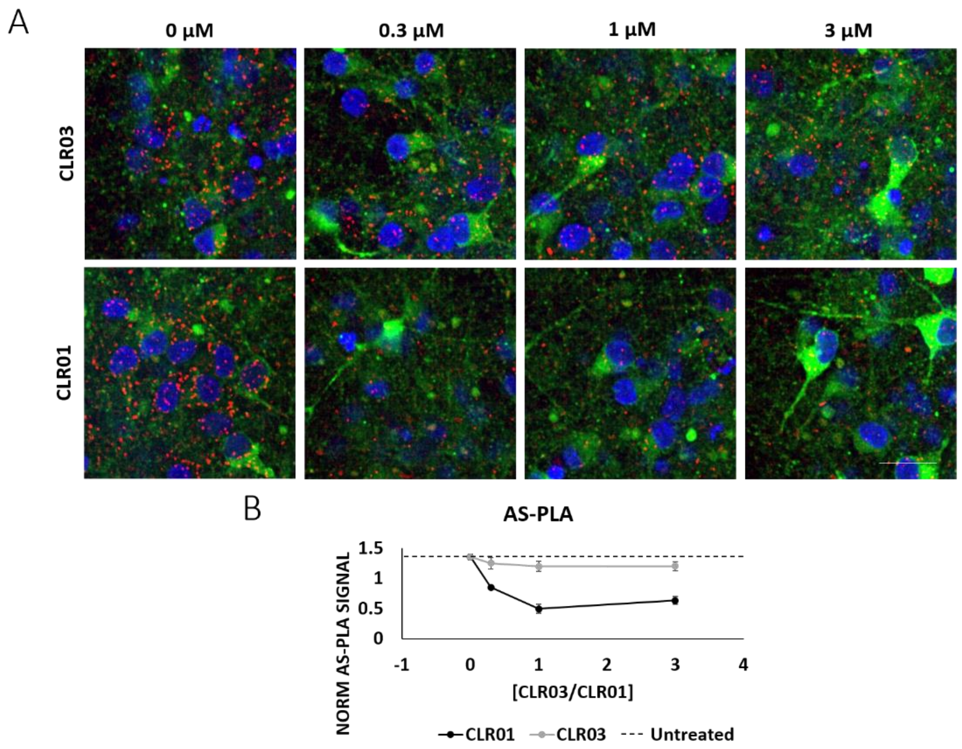

Supplementary Fig. 5. CLR01 reduces oligomeric α-syn in LB extract-treated iPSC dopaminergic cultures. **(A and B)** Representative immunofluorescence images and quantification of AS-PLA (red) in dopaminergic cultures exposed to LB extracts which had been previously treated with increasing concentrations of CLR03 or CLR01. Quantification of indicated parameters of 3 independent control cell lines. Scale bars = 25 μm. For all appropriate panels data are presented as mean values +/- SEM.

Supplementary Fig. 6 CLR01 reduces α-syn transport *in vitro*.

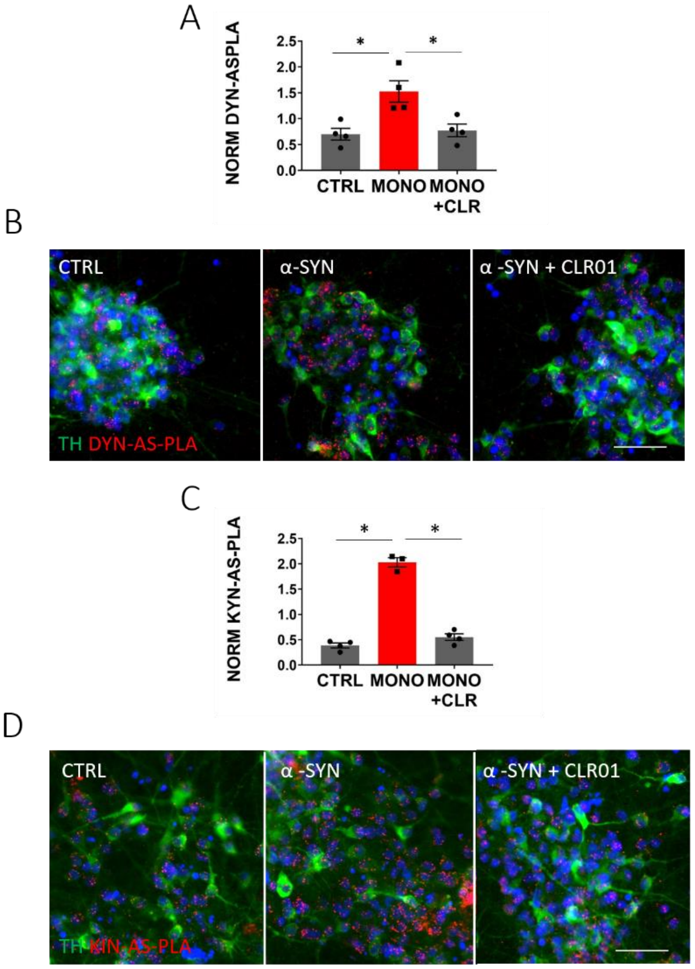

Supplementary Fig. 6. CLR01 reduces α-syn transport *in vitro*. **(A and B)** Representative immunofluorescence images and quantification of α-syn-dynein-PLA signal (red) in dopaminergic cultures (TH+ cells in green). Cells were treated α-syn monomer in order to determine whether α-syn was able to interact with the retrograde transport protein dynein. Scale bars = 50 μm. **(C and D)** Representative immunofluorescence images and quantification of α-syn-kinesin-PLA signal (red) in dopaminergic cultures (TH+ cells in green). Cells were treated α-syn monomer in order to determine whether α-syn was able to interact with the anterograde transport protein kinesin. A: $F (2, 9) = 8.905$, $p = 0.0082$, $p = 0.0140$. C: $F (2, 8) = 163.2$, $p < 0.0001$, $p < 0.0001$. * $p < 0.05$, ** $p < 0.01$, *** $p < 0.001$. **(A-D)** Quantification of indicated parameters of 4-5 independent control cell lines, through One-Way ANOVA (Sidak). For all appropriate panels data are presented as mean values +/- SEM.

Supplementary Fig. 7 Microglial morphology.

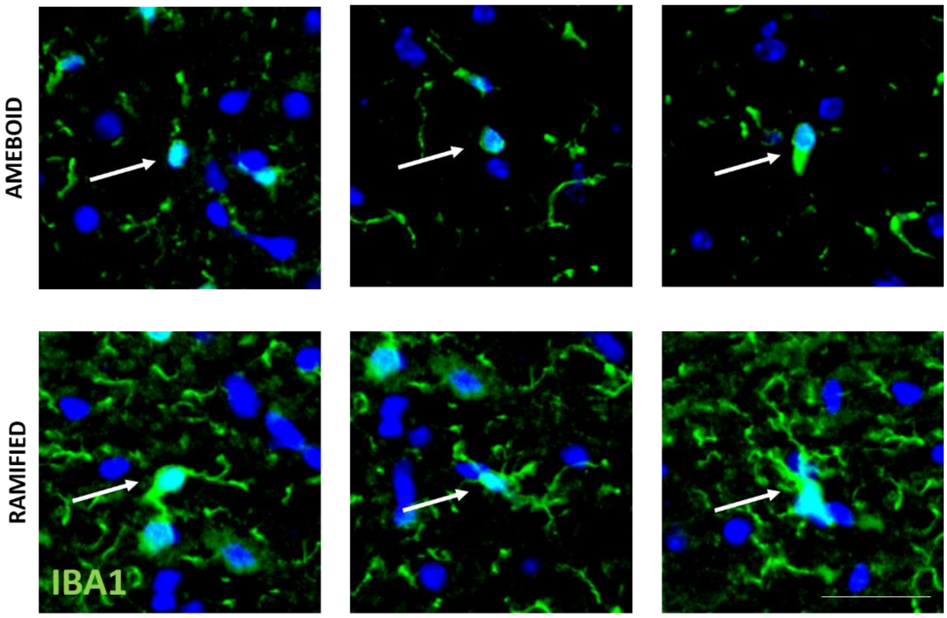

Supplementary Fig. 7. Microglial morphology. Microglial cells were blindly classified as ameboid or ramified depending on whether they extended processes into their vicinity. In this figure we show examples of ameboid and ramified morphologies taken from Iba1 IF, n = 3-4 animals per group. Scale bars = 50 µm.

Supplementary Fig. 8 CLR01 brain penetration and *in silico* modelling of brain pharmacokinetics.

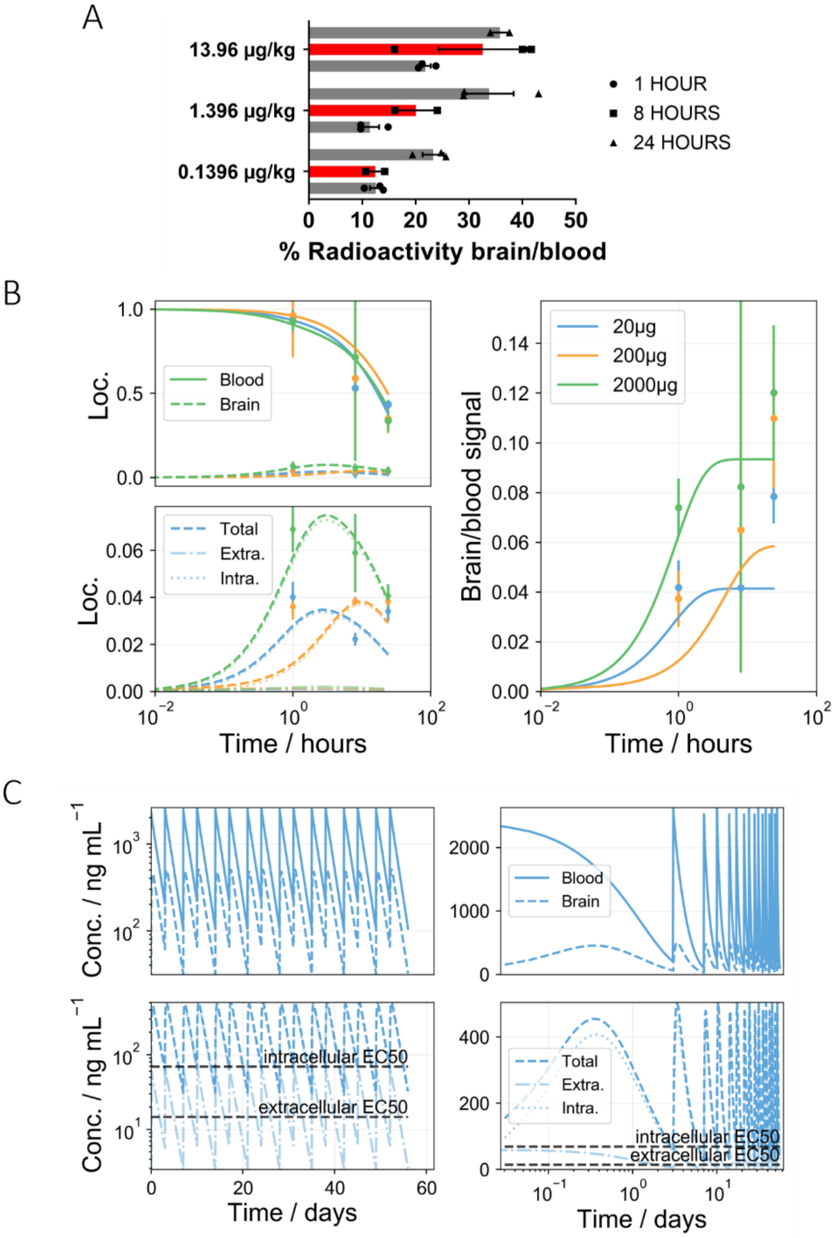

Supplementary Fig. 8. CLR01 brain penetration and *in silico* modelling of brain pharmacokinetics. (A) Tritium-labeled CLR01 was sub-cutaneously administered to animals at the indicated doses. After 1, 8 or 24 h blood and brain were harvested and radioactivity levels of the samples were measured. The percentage of the radioactivity detected in the brain relative to that of the blood was calculated in order to produce the final graph. N = 3 for all groups except for time point 8 h and 0.1396 and 1.396 µg/kg dose (n = 2), and for time point 24 h dose 13.96 µg/kg (n = 2). (B) Fitted localization of the compound in blood and brain (top left), total, extracellular and intracellular (bottom left) and fittings of brain to blood signal over time at different doses, n = 3 independent animals. (C) Predicted concentration after 16 sub-cutaneous doses over two months at 40 µg/kg/day in blood and brain (both intracellular and extracellular). For all appropriate panels data are presented as mean values +/- SD. Loc: localization, intra: intracellular, extra: extracellular, conc: concentration.

Supplementary Fig. 9 Schematic representation of CLR01 treatment in animal cohorts in this study.

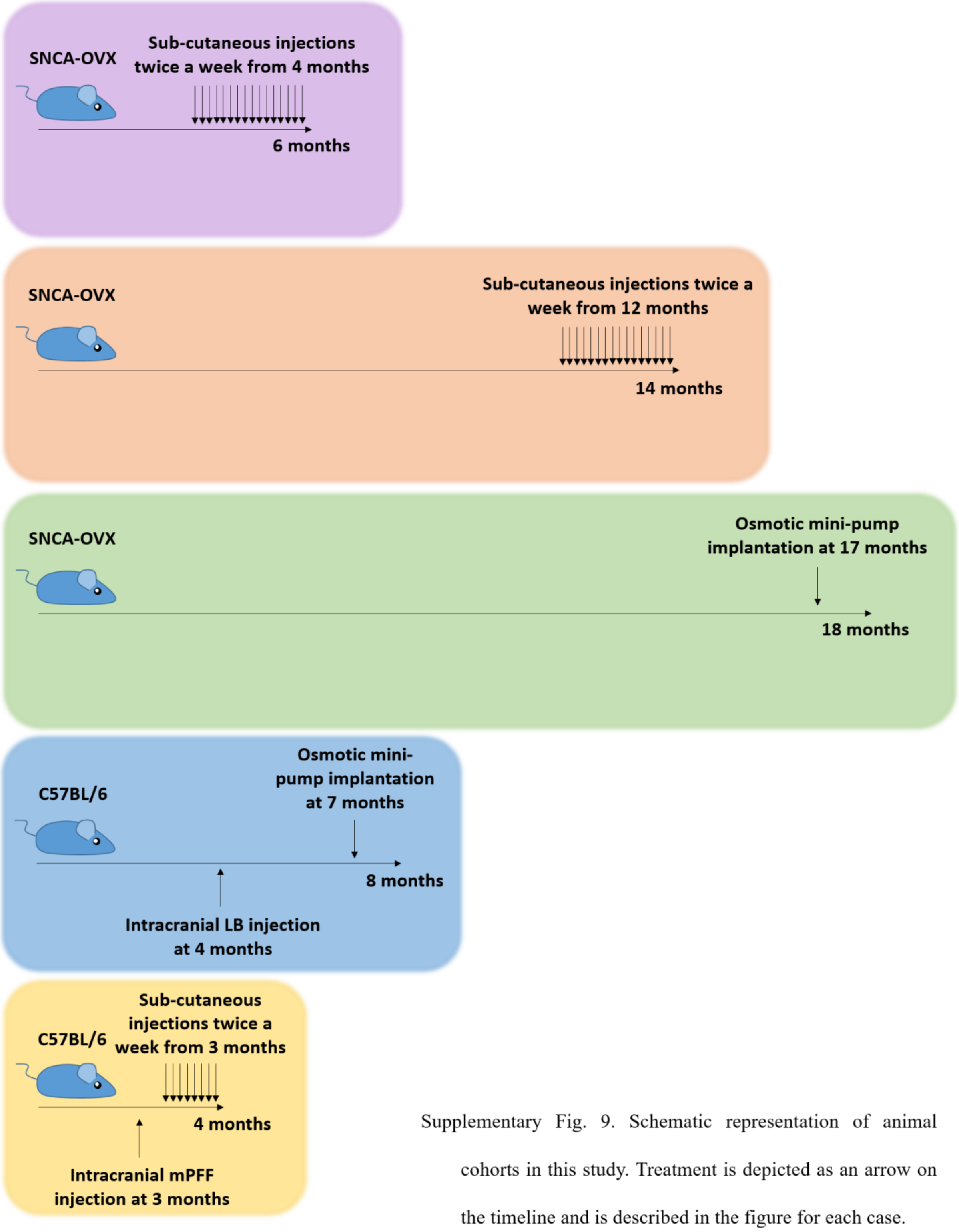

Supplementary Fig. 9. Schematic representation of animal cohorts in this study. Treatment is depicted as an arrow on the timeline and is described in the figure for each case.

Supplementary Fig. 10 CLR01 effects at 6 months.

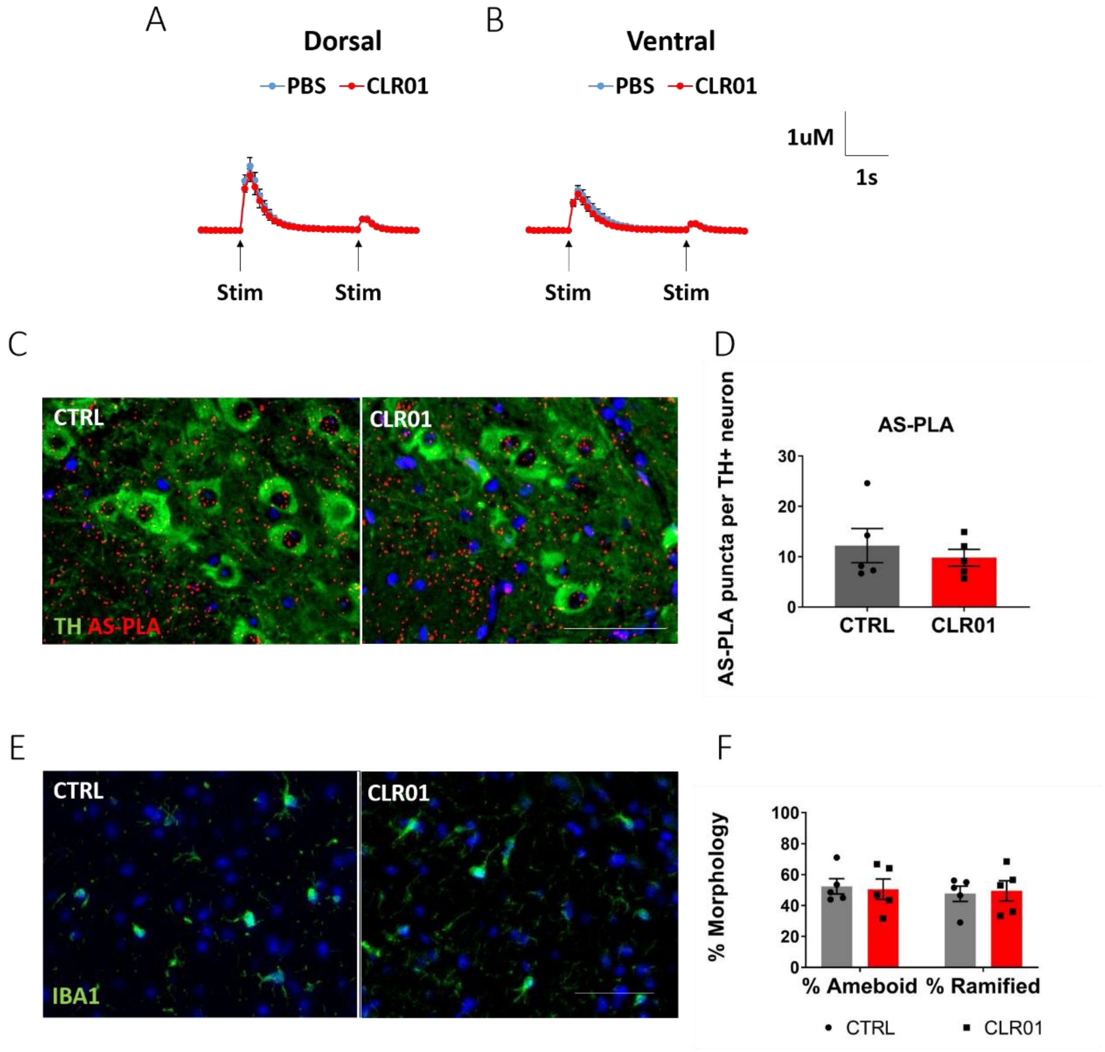

Supplementary Fig. 10. CLR01 effects on *SNCA-OVX* mice at 6 months of age. **(A and B)** Traces of FCV analysis of dorsal and ventral striatal dopamine release in animals treated with 40 µg/kg/d PBS/CLR01 for two months at 4 months of age. **(C and D)** Representative images and quantification of AS-PLA puncta per TH+ cell. Scale bars = 50 µm. **(E and F)** Representative images and quantification of microglial morphology (expressed as a % from total). Scale bars = 25 µm. **(A-F)** All parameters were analyzed using a two-tailed t-test n = 5/5 independent animals. For all appropriate panels data are presented as mean values +/- SEM. Stim: stimulus.

Supplementary Fig. 11 CLR01 effects on the behaviour of SNCA-OVX mice at 12 months.

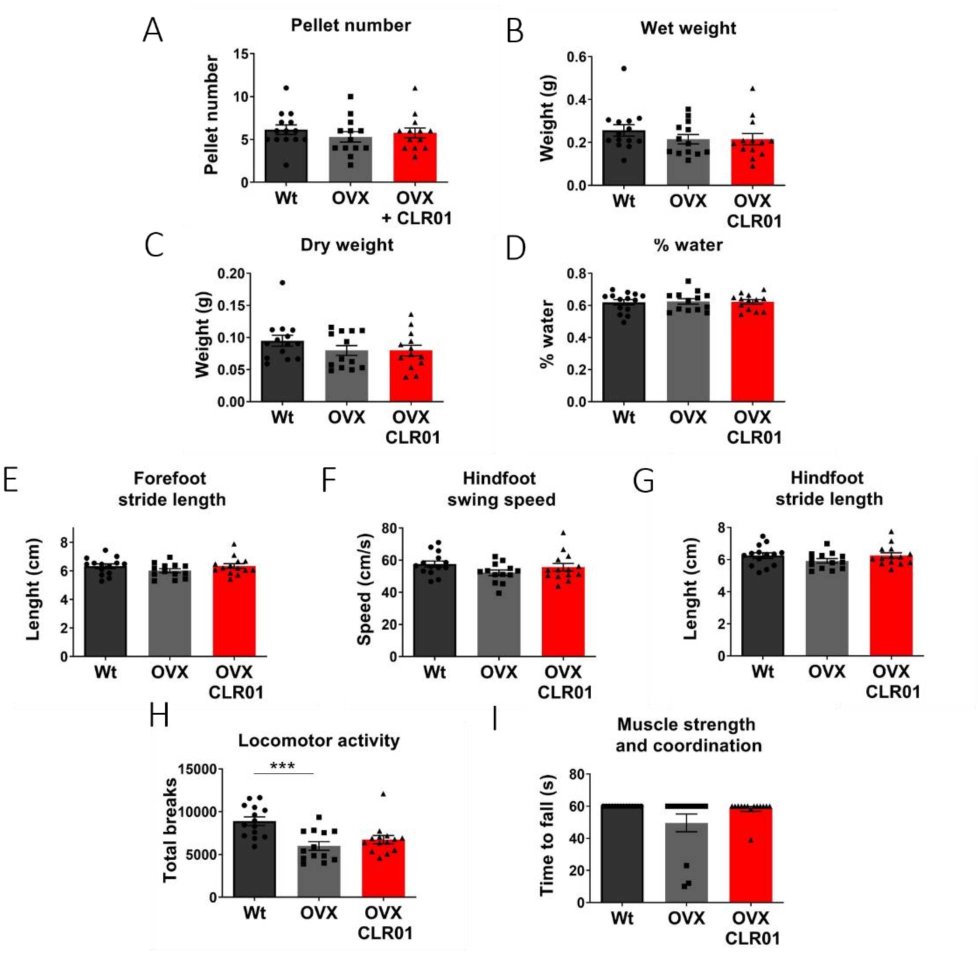

Supplementary Fig. 11. CLR01 effects on the behavior of *SNCA-OVX* mice at 12 months of age. **(A-D)** Quantification of stool collection analysis after sub-cutaneous implant of an osmotic mini-pump. **(E-G)** Quantification of catwalk gait analysis, **(H)** locomotor analysis (LMA) and **(I)** muscle strength and coordination. All parameters were analyzed using a One-way ANOVA (Sidak) n = 11-14 independent animals. H: F (2, 38) = 9.515, p = 0.0003. For all appropriate panels data are presented as mean values +/- SEM.

Supplementary Fig. 12 CLR01 effects on the behaviour of Wt versus SNCA-OVX mice at 12 months.

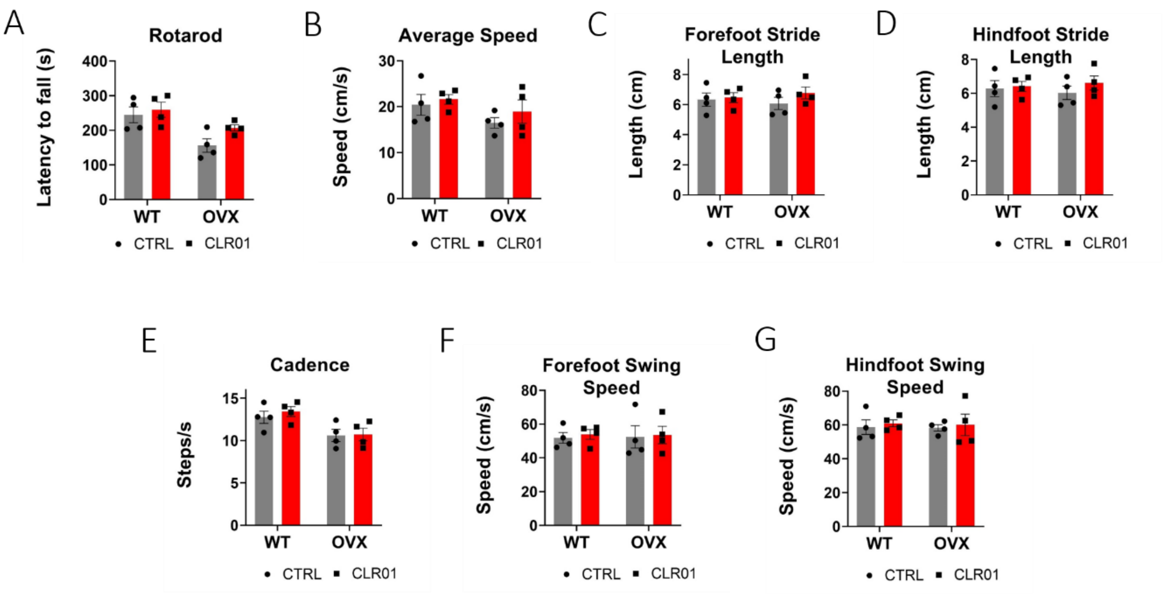

Supplementary Fig. 12. The effect of CLR01 on the behaviour of Wt versus *SNCA*-OVX mice at 12 months. Quantification and analysis of **(A)** rotarod and **(B-G)** catwalk gait. n = 4. For all appropriate panels data are presented as mean values +/- SEM.

Supplementary Fig. 13 CLR01 effects on the behaviour of SNCA-OVX mice at 18 months.

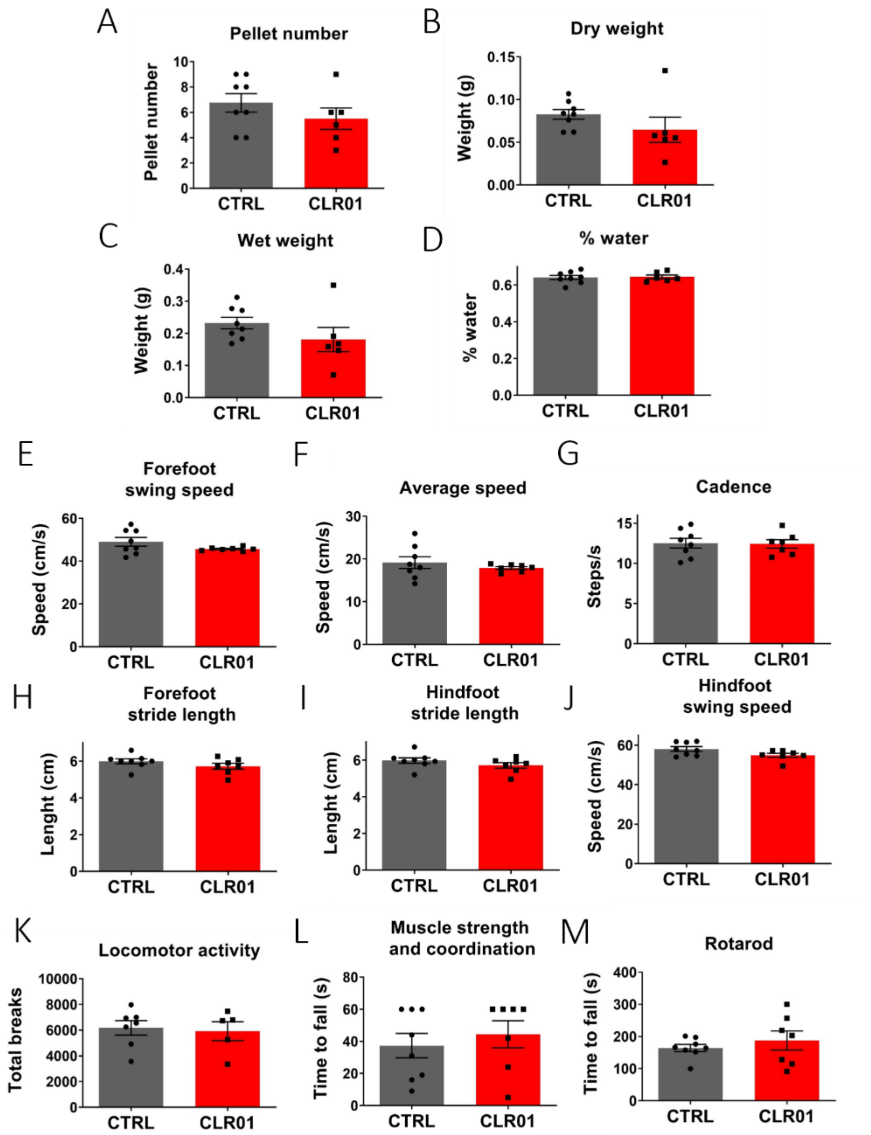

Supplementary Fig. 13. CLR01 effects on the behavior of *SNCA-OVX* mice at 18 months of age. **(A-D)** Quantification of stool collection analysis after sub-cutaneous implant of an osmotic mini-pump. **(E-J)** Quantification of catwalk gait analysis, **(K)** locomotor analysis (LMA), **(L)** muscle strength and coordination, and **(M)** rotarod. **(A-M)** All parameters were analyzed using a two-tailed t-test n = 7-8 independent animals. For all appropriate panels data are presented as mean values +/- SEM.

Supplementary Fig. 14 CLR01 effects on glial cells and molecular markers.

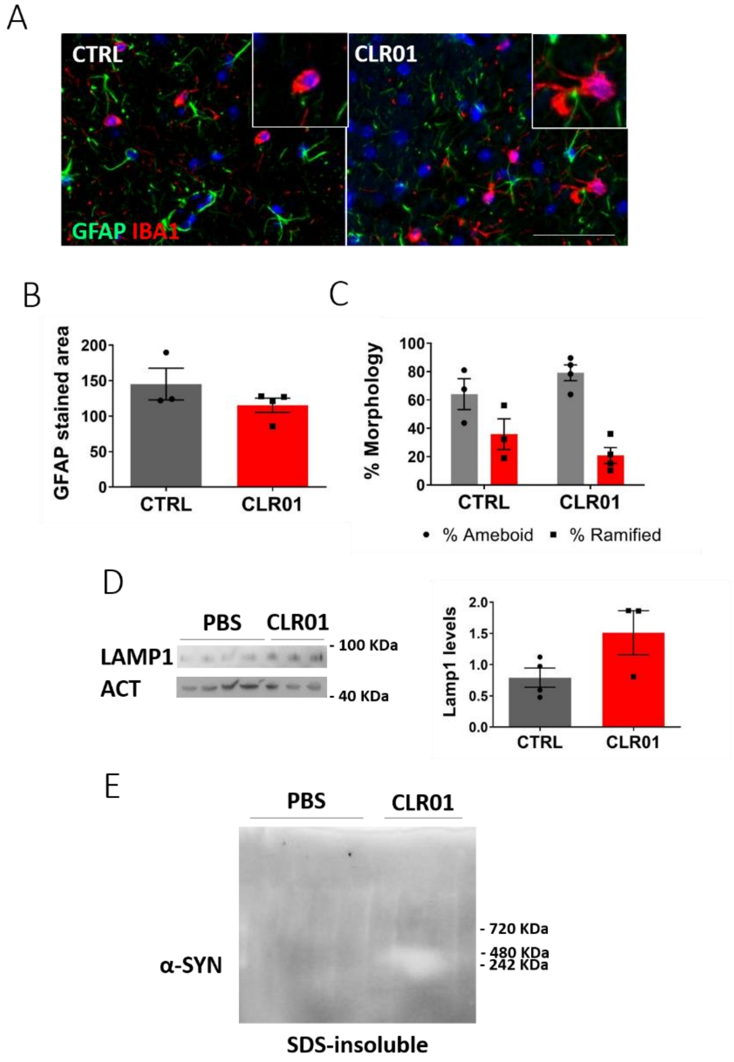

Supplementary Fig. 14. CLR01 effects on aggregation in glial cells and molecular markers in *SNCA-OVX* mice. **(A-C)** Representative images and quantification of GFAP stained area per field and microglial morphology (expressed as a % from total). Scale bars = 50 µm. **(D)** Western blot and quantification of Lamp1 **(E)** Native western blot of midbrain SDS-insoluble α-syn. All panels analyzed using a one tailed Mann-Whitney U-test n = 3-4 independent animals. * $p < 0.05$. n = 3-4. AV: average, CLR: CLR01. For all appropriate panels data are presented as mean values +/- SEM.

Supplementary Fig. 15 CLR01 reduces α-syn aggregation in a mPPF injection model.

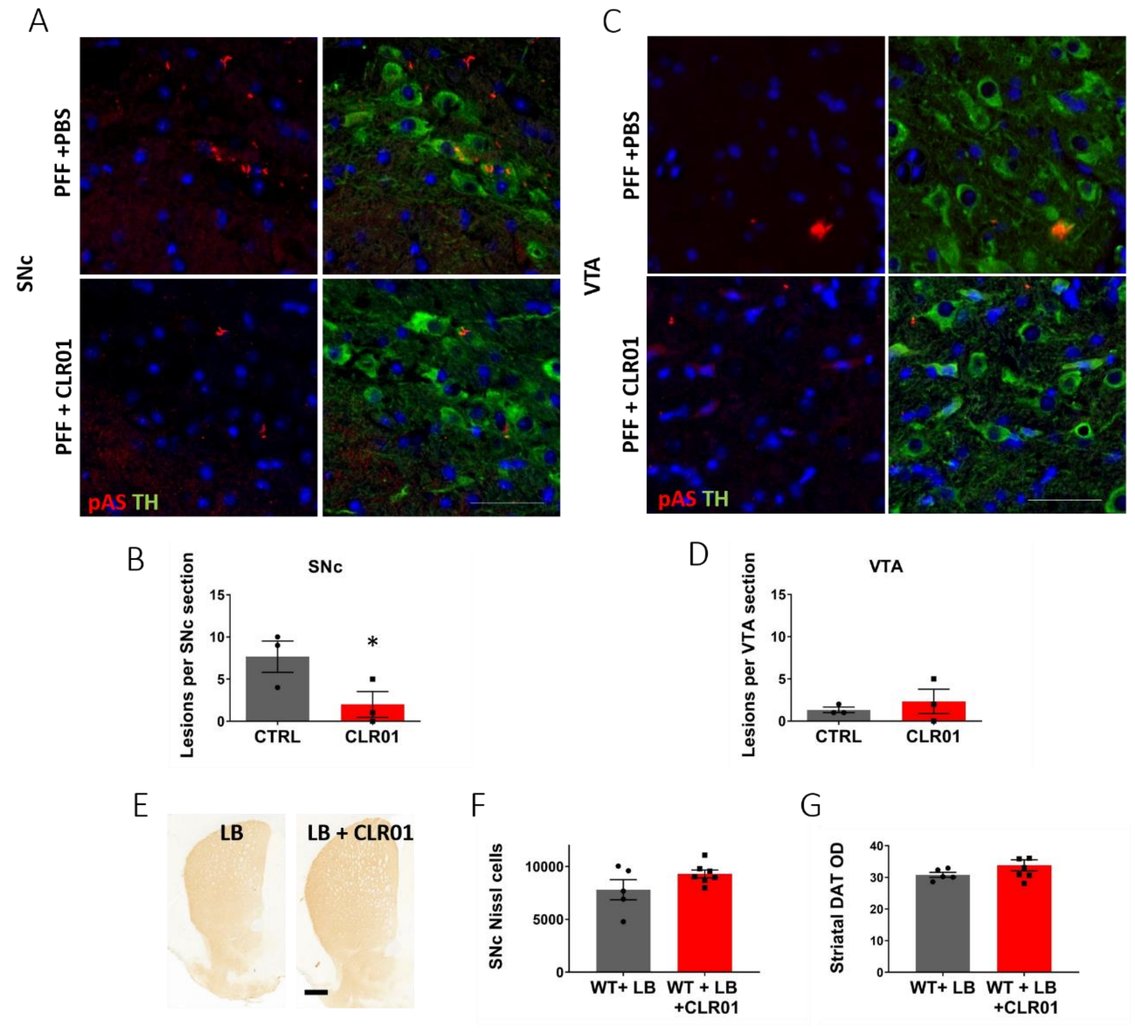

Supplementary Fig. 15. CLR01 reduces α-syn aggregation in a mPFF injection model. **(A)** Representative images of phospho-α-syn (pAS) and TH in the SNc after sub-cutaneous injection of 400 μg/kg/d CLR01 or PBS. Scale bars = 100 μm. **(B)** Corresponding quantification of lesions per SNc analyzed blind to genotype counting all Lewy-like inclusions in TH+ cells and analyzed using a one-tailed t-test n = 3 independent animals. $p = 0.0389$ **(C)** Representative images of phospho-α-syn (pAS) and TH of in the VTA after sub-cutaneous injection of 400 μg/kg/d CLR01 or PBS. Scale bars = 100 μm. **(D)** Corresponding quantification of average lesions per VTA analyzed blind to genotype counting all Lewy-like inclusions in TH+ cells and analyzed using a t-test n = 3 independent animals. * $p < 0.05$. CLR: CLR01, AV: average, VTA: ventral tegmental area, SNc: substantia nigra pars compacta. **(E)** Representative photomicrographs of DAT-immunostained striatum in LB-inoculated mice after osmotic mini-pump implantation for CLR01 (or PBS) delivery (40μg/kg/hr). Scale bar 1 mm. **(F)** Stereological cell counts of SNpc nissl-stained cells in LB-inoculated mice, at 3 months post-LB inoculation. **(G)** Optical densitometry of striatal DAT immunoreactivity in LB-inoculated mice, at 3 months post-LB inoculation. **(E-G)** analyzed with a two-tailed Mann-Whitney U-test *: $p< 0.05$. n=5-7 independent LB and LB + CLR01-treated animals. For all appropriate panels data are presented as mean values +/- SEM.

Supplementary Fig. 16 CLR01 structure and α-syn aggregates.

A

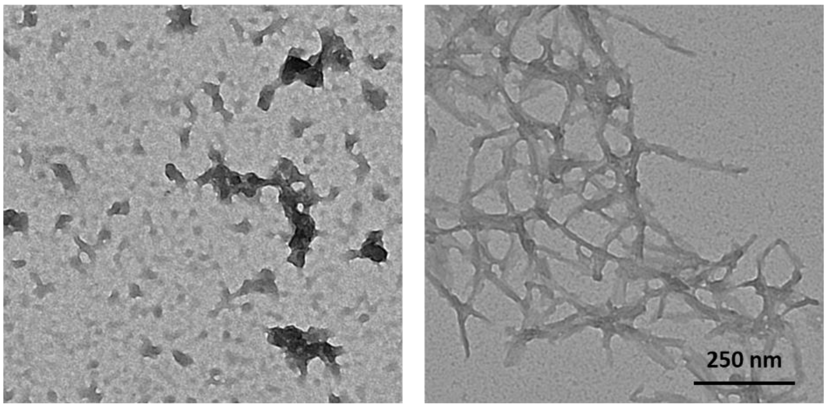

B

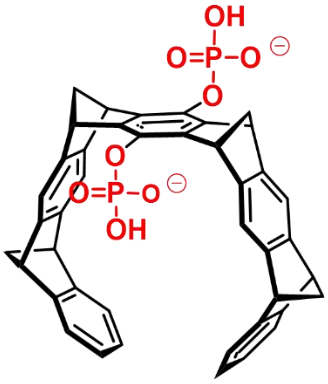

Supplementary Fig. 16. CLR01 structure and α-syn aggregates. **(A)** Example of α-syn aggregates used for *in vitro* and *in vivo* experiments; human oligomers (left) and mPFF (right). The same batch (n = 1) was used for all experiments for consistency. Scale bars = 250 nm. **(B)** Chemical structure of CLR01.

Supplementary table

Supplementary Table 1 CLR01 pharmacokinetic parameters.

	AUC (ngh/ml)	Time averaged AUC (ng/ml)	Cmax (ng/ml)	Clearance rate (h)	Time above EC50 (%)
**Extracellular (AV/SD)**	1.4	100	230	0.02	81
**Intracellular (AV/SD)**	3.2	240	510	6	93

Supplementary Table 1. Pharmacokinetic parameters of CLR01. All parameters were calculated based on our *in silico* modeling. AUC: area under the curve, Cmax: maximum concentration, AV: average, SD: standard deviation.

Supplementary Methods

iPSC-derived dopaminergic cultures

iPSCs were dissociated to single cells and plated at a density of 150,000 cells/cm2 on Geltrex (Life Technologies) and cultured until confluent. Cells were then grown 11 days in knockout serum replacement medium (KSR) containing KO DMEM (Life Technologies), 15% knockout serum replacement (Life Technologies), 2 mM L-glutamine (Life Technologies) and 10 mM β-mercaptoethanol (Sigma-Aldrich). KSR medium was gradually shifted to NNB medium containing Neurobasal medium, 0.5X N2, 0.5X B27 and 2 mM L-glutamine (Life Technologies) starting on day 5 of differentiation. iPSCs were patterned for 11 days to become ventral midbrain precursors cells with different combinations of LDN193189 (100nM, Sigma-Aldrich), SB431542 (10 mM, Tocris), SHH C24II N-terminus (100 ng ml-1, Bio-Techne), Purmorphamine (2 mM, Millipore), FGF8a (100 ng ml-1, Bio-Techne) and CHIR99021 (3 mM, Tocris). On day 11, media was changed to NB medium containing Neurobasal medium, B27 and 2 mM L-glutamine (Life Technologies) supplemented with CHIR (until day 13) and with BDNF (brain-derived neurotrophic factor, 20n ml-1; Peprotech), ascorbic acid (0.2 mM, Sigma-Aldrich), GDNF (glial cell line-derived neurotrophic factor, 20 ng ml-1; Peprotech), TGFβ3 (transforming growth factor type β3, 1 ng ml-1; Peprotech), dibutyryl cAMP (0.5mM; Sigma-Aldrich), and DAPT (10mM; Abcam) for 9 days. On day 20, cells were dissociated using Accutase (Life Technologies) and replated at the cell density of 300,000 cells/cm2 on Geltrex in final differentiation medium (NB supplemented with BDNF, GDNF, TGFb3, DAPT, dbcAMP and ascorbic acid in the above mentioned concentrations). On day 22, cells were treated for an hour with 1 μg ml-1 of Mitomycin C (Life Technologies) in NB medium. Cell were then washed and cultured in final differentiation medium, with half-medium changes every Monday, Wednesday and Friday until experiments were performed.

Modelling methodology

1.1 Mathematical model of blood kinetics

To fit data describing the clearance of CLR01 from the blood after intravenous or subcutaneous delivery, three different functions were proposed: exponential decay,

$N_0 e^{-kt},$ (Supplementary equation 1)

the sum of two exponentials,

$N_0 (e^{k_1 t} + a e^{k_2 t}),$ (Supplementary equation 2)

and a Gaussian,

$N_0 e^{-(t-\mu)^2/\sigma^2}.$ (Supplementary equation 3)

Each function was fit to data. The best function was selected by the sum of least squares from the data, its R^2 value and the number of parameters required.

1.2 Mathematical model of cellular uptake

1.2.1 Model definition

To fit data describing cellular uptake of fluorescently labelled CLR01, we constructed a two-compartment model without physiological details such of blood or brain tissue,

$$\frac{\mathrm{d}N_I}{\mathrm{dt}} = \gamma\left(\theta\frac{V_I}{V_E}N_E - N_I\right),$$

$$\frac{\mathrm{d}N_E}{\mathrm{dt}} = \gamma\left(N_I - \theta\frac{V_I}{V_E}N_E\right), \qquad \text{(Supplementary equation 4)}$$

where N_I and N_E are the number of molecules in the intracellular and extracellular compartments, V_I and V_E are the volume of the intracellular and extracellular compartments, γ is the transfer rate between those compartments and θ is the equilibrium ratio of the intracellular 3D intensity (counts/time/volume) to the extracellular 3D intensity.

1.2.2 Conversion between 2D and 3D

Fluorescence microscopy images are 2D, but the physical exchange of molecules between the cell and its surroundings occurs in 3D space. Supplementary table 2 summarises how the area, number of cells and intensities in the 2D images are assumed to scale to their 3D counterparts. When Supplementary equation 4 is at equilibrium,

$$\frac{N_I}{N_E} = \theta\frac{V_I}{V_E}. \qquad \text{(Supplementary equation 5)}$$

From Supplementary table 2, we have that

$$\frac{I_3}{E_3} = \frac{V_I I_2}{dA_I}\frac{dA_E}{V_E E_2} = \frac{V_I}{V_E}\frac{A_E}{A_I}\frac{I_2}{E_2}, \qquad \text{(Supplementary equation 6)}$$

where $I_3 := N_I$ and $E_3 := N_E$ are the number of molecules in the intracellular and extracellular compartments in 3D space, I_2 and E_2 are the numbers captured in the 2D images, A_I and A_E are the intracellular and extracellular areas in the image and d is the depth of the imaging area. Combining Supplementary equations 5 and 6 allows the ratio of 2D intensities to be written as,

$$\frac{I_2}{E_2} = \theta\frac{A_I}{A_E}, \qquad \text{(Supplementary equation 7)}$$

so the ratio of the intracellular intensity to the extracellular intensity, θ, is the same for 2D intensities and 3D "intensities". This allows comparison of 2D data with the model's 3D output and calculation of the parameters θ and γ without knowing the image depth *d*.

Non-dimensional constants	
Number of cells	n
Depth of imaging area	d
2D	3D
Total image area A	Total volume $V = A^{3/2}$
Total intracellular area A_I	Total intracellular volume $V_I = n(\frac{A_I}{n})^{3/2}$
Extracellular area $A_E = A - A_I$	Extracellular volume $V_E = V - V_I$
Intracellular molecule density σ_I	Intracellular molecule density $\rho_I = \sigma_I / d$
Extracellular molecule density σ_E	Extracellular molecule density $\rho_E = \sigma_E / d$
Intracellular molecule count $I_2 = \sigma_I A_I = \rho_I d A_I$	Intracellular molecule count $I_3 = \rho_I V_I = \frac{V_I}{dA_I} I_2$
Extracellular molecule count $E_2 = \sigma_E A_E = \rho_E d A_E$	Extracellular molecule count $E_3 = \rho_E V_E = \frac{V_E}{dA_E} E_2$

Supplementary Table 2: Scaling of physical characteristics between a 3D system and quantities observed in a 2D image.

1.2.3 Solution to equations

Supplementary equation set 4 can be solved exactly, assuming that N_I $(t = 0) = 0$ and N_I (t) + $N_E(t) = T_0$:

$$N_I(t) = \frac{\theta V_I T_0}{\theta V_I + V_E}\left(1 - e^{-\gamma\left(\theta\frac{V_I}{V_E}+1\right)t}\right)$$

$$N_E(t) = \frac{V_E T_0}{\theta V_I + V_E}\left(1 + \theta\frac{V_I}{V_E} e^{-\gamma\left(\theta\frac{V_I}{V_E}+1\right)t}\right), \qquad \text{(Supplementary equation 8)}$$

which in turn can be used to create initial estimates for parameters θ and γ:

$$\theta_0 = \frac{N_I(t_\infty)/V_I}{N_E(t_\infty)/V_E}$$

$$\gamma_0 = \frac{\log\left(\frac{T_0}{T_0 - N_I(t_1)\left(1 + \frac{V_E}{\theta V_I}\right)}\right)}{\left(\theta\frac{V_I}{V_E} + 1\right)t_1}, \qquad \text{(Supplementary equation 9)}$$

where t_∞ is the last available time at which data is available, and t_1 is the first time after zero at which data is available.

1.2.4 Application of data

Recorded data takes the form of two-dimensional intensities, $D_I = I_2/A_I$ and $D_E = E_2/A_E$. The known intracellular and extracellular areas are used to calculate the total two-dimensional luminosity, and the equations in Supplementary table 2 are used to calculate the three-dimensional luminosity in terms of a dummy image depth d. The three-dimensional luminosity is assumed to be proportional to the number of molecules in the intracellular regions N_I and N_E. Normalising the data and the model output by the total luminosity and total number of simulated molecules allows the data to be plotted on the same axes. Hence, θ and γ and may be estimated without knowing how many molecules a unit of luminosity corresponds to, or the image depth d.

1.3 Mathematical model of physiologically based pharmacokinetics

1.3.1 Model definition

To fit the measured localisation of labelled CLR01 in the brain and blood, the following physiologically based model was constructed,

$$V_{\text{brain}}\frac{\mathrm{d}C_{\text{brain}}}{\mathrm{dt}} = B(C_{\text{heart}} - C_{\text{brain}}),$$

$$\tilde{V}_{\text{brain}}\frac{\mathrm{d}\tilde{C}_{\text{brain}}}{\mathrm{dt}} = eB\left(C_{\text{brain}} - \mu\tilde{C}_{\text{brain}}\right),$$

$$V_{\text{heart}}\frac{\mathrm{d}C_{\text{heart}}}{\mathrm{dt}} = B(C_{\text{brain}}(1-e) - C_{\text{heart}}) + e\mu B\tilde{C}_{\text{brain}} - dV_{\text{heart}}C_{\text{heart}},$$

(Supplementary equation 10)

where V denotes vascular volume, $\tilde{V}$ interstitial volume and C concentration of CLR01 in the named compartment. The label “heart” refers to blood everywhere but in the brain. B is the blood flow to the brain. e is the proportion of CLR01 passing through the vasculature of the brain that crosses the blood-brain barrier. μ is the equilibrium ratio of concentrations in the brain interstitial space to the vascular space. d is the rate of loss of signal from the blood. Supplementary equation set 10 can be expanded to include intracellular and extracellular compartments as in Supplementary equation set 4:

$$V_{\text{v}}\frac{\mathrm{d}C_{\text{brain-v}}}{\mathrm{dt}} = B(C_{\text{heart}} - C_{\text{brain-v}}),$$

$$\tilde{V}_{\text{e}}\frac{\mathrm{d}\tilde{C}_{\text{brain-e}}}{\mathrm{dt}} = eB\left(C_{\text{brain-v}} - \mu\tilde{C}_{\text{brain-e}}\right) + \gamma\left(\tilde{N}_{\text{brain-i}} - \theta\frac{\tilde{V}_i}{\tilde{V}_e}\tilde{N}_{\text{brain-e}}\right),$$

$$\tilde{V}_{\text{i}}\frac{\mathrm{d}\tilde{C}_{\text{brain-i}}}{\mathrm{dt}} = \gamma\left(\theta\frac{\tilde{V}_i}{\tilde{V}_e}\tilde{N}_{\text{brain-e}} - \tilde{N}_{\text{brain-i}}\right),$$

$$V_{\mathrm{heart}}\frac{\mathrm{d}C_{\mathrm{heart}}}{\mathrm{dt}} = B(C_{\text{brain-v}}(1-e) - C_{\mathrm{heart}}) + e\mu B\tilde{C}_{\text{brain}-\mathrm{e}} - dV_{\mathrm{heart}}C_{\mathrm{heart}},$$

(Supplementary equation 11)

where $\tilde{N} = \tilde{C}\tilde{V}$, $C_{\text{brain-v}}$ is the brain's vascular compartment, $C_{\text{brain-e}}$ is its extracellular compartment and $C_{\text{brain-i}}$ is its intracellular compartment. If blood kinetics are assumed to be fast compared to the transfer of CLR01 across the brain-brain barrier, the first Supplementary equations in sets 10 and 11 rapidly reach equilibrium and $C_{\mathrm{heart}} = C_{\mathrm{brain}} := C_{\text{brain-v}} = C$, allowing the system to be simplified to,

$$\tilde{V}_{\mathrm{e}}\frac{\mathrm{d}\tilde{C}_{\mathrm{e}}}{\mathrm{dt}} = eB\left(C - \mu\tilde{C}_{\mathrm{e}}\right) + \gamma\left(\tilde{N}_{\mathrm{i}} - \theta\frac{\tilde{V}_{\iota}}{\tilde{V}_{e}}\tilde{N}_{e}\right),$$

$$\tilde{V}_{\mathrm{i}}\frac{\mathrm{d}\tilde{C}_{\mathrm{i}}}{\mathrm{dt}} = \gamma\left(\theta\frac{\tilde{V}_{\iota}}{\tilde{V}_{e}}\tilde{N}_{e} - \tilde{N}_{i}\right),$$

$$V_{\mathrm{blood}}\frac{\mathrm{d}C}{\mathrm{dt}} = eB\left(\mu\tilde{C}_{\mathrm{e}} - C\right) - dV_{\mathrm{heart}}C,$$

(Supplementary equation 12)

where $V_{\mathrm{blood}} := V_{\mathrm{brain}} + V_{\mathrm{heart}}$ is the total blood volume, and $\tilde{C}_{\text{brain-e}}$ and $\tilde{C}_{\text{brain-i}}$ have been relabelled as $\tilde{C}_{\mathrm{e}}$ and $\tilde{C}_{\mathrm{i}}$ for clarity.

1.3.2 Initial conditions

The initial conditions of these systems are the total number of molecules in a dose of CLR01, assumed to initially be entirely within the blood compartment. Additional doses can be introduced to the same compartment at later, defined time-points.

1.3.3 Estimates for unknown parameters

If e and μ are the only unknown parameters, estimates may be formed in terms of the blood decay rate d, physiological parameters and known data for N. At early times, where it is assumed that return of CLR01 from the 3 brain can be ignored ($\mu = 0$), e can be estimated with,

$$eB \approx V_{\mathrm{blood}}\frac{\log\left(\frac{N_{\mathrm{blood}}(0)}{N_{\mathrm{blood}}(t_1)}\right)}{t_1} - dV_{\mathrm{heart}},$$

(Supplementary equation 13)

where t_1 is the first time point after zero for which data exists. At late times, it can be assumed that the vascular and brain extracellular compartments have reached equilibrium and

$$\mu \approx \frac{C(t_\infty)}{\tilde{C}_{\mathrm{e}}(t_\infty)}.$$

(Supplementary equation 14)

If the blood loss rate d and/or the total amount of CLR01 at the initial time point are also unknown, then a less accurate estimate for e is given by,

$$e \approx \frac{V_{\mathrm{blood}}}{B}\frac{\log\left(\frac{N(t_1)}{N(t_2)}\right)}{t_2 - t_1}\frac{\tilde{N}_e(t_2)}{N(t_1) - N(t_2)}\left(\frac{N(t_2)}{N(t_1)}\right)^{\frac{t_1}{t_2 - t_1}},$$

(Supplementary equation 15)

where t_1 and t_2 are the two earliest time points after zero for which data exists, and where it is assumed that return from the brain can be ignored ($\mu = 0$) until time t_2. An initial guess for the blood loss rate d is also obtained under these assumptions,

$$d \approx \frac{V_{\text{blood}}}{V_{\text{heart}}} \frac{\log\left(\frac{N(t_1)}{N(t_2)}\right)}{t_2 - t_1} \left(1 - \frac{\tilde{N}_e(t_2)}{N(t_1) - N(t_2)} \left(\frac{N(t_2)}{N(t_1)}\right)^{\frac{t_1}{t_2 - t_1}}\right).$$

(Supplementary equation 16)

The estimates in Supplementary equations 14, 15 and 16 are used to initialise fitting procedures of Supplementary equation set 12 to pharmacokinetic data.

1.4 Mathematical model of dosing regimen

The experimental procedure described above is modelled mathematically using Supplementary equation set 12 and assuming that a (140 µg/kg x 34.3 g = 4.8 µg) dose of CLR01 is added to the blood compartment N_{blood} at times $t \in$ {0, 3, 7, 10, 14, 17, 21, 24, 28, 31, 35, 38, 42, 45, 49, 52} days. Parameters of interest were calculated as follows:

- Area under curve (average over brain): A numerical integration, using Simpson's rule, of the sum of the amount of material in the intracellular and extracellular compartments of the brain, divided by the brain's total volume.
- Area under curve (intracellular only): A numerical integration, using Simpson's rule, of the intracellular compartment of the brain, divided by the intracellular volume.
- Time-averaged areas under curve: The values are divided by the maximum simulation time, which is 56 days.
- Cmax (average over brain): The largest value across time of the sum of the amount of material in the intracellular and extracellular compartments of the brain, divided by the total brain volume.
- Cmax (intracellular only): The largest value across time of the amount of material in the intracellular compartment of the brain, divided by the intracellular volume.
- Half-life of compound in blood: The predicted value for the blood loss rate, d.
- Half-life of compound in brain extracellular space: The predicted value for the rate of exit from the extracellular compartment, $e\mu B/\tilde{V}_e$. Note that this value is only valid when there is no compound in the blood.
- Half-life of compound in brain intracellular space: The predicted value for the rate of exit from the intracellular compartment, γ. Note that this value is only valid when there is no compound in the extracellular space.

- Time above EC50 (averaged across brain): The set of numerical time points t_i for which the average concentration of material in the intracellular and extracellular compartments of the brain is above a given EC50 is identified. The difference in time between these points and the next interval, $\delta_i = t_i + 1 - t_i$ is calculated. The time for which the concentration is above this EC50 is assumed to be the sum of these time intervals $\sum_i \delta t_i$.

- Time above EC50 (intracellular only): As above, but only considering the concentration in the intracellular compartment of the brain.

1.5 Parameter fitting methods

Three different parameter fitting methods were used. SciPy's curve fit and basinhopping [1], and a hierarchical approach using the Haario Bardenet Adaptive Covariance Monte Carlo method in PINTS [2], a Python library for Markov Chain Monte Carlo (MCMC) methods. In each case, initial guesses were made to parameters as defined in section 1.3.3 and reasonable estimates were made of the maximum and minimum values of each parameter, to provide boundaries and a uniform prior for the MCMC method. Variates of the model were scored according to the least-squares-difference between the model output and the data.

1.6 Physiological parameterisation

The blood flow to the brain, total volume of the brain, blood volume of the brain and total blood volume were estimated using data from Brown et al and Shah et al [3, 4]. Cardiac output is assumed to scale as (animal age)$^{0.75}$ [3] and brain volume and total blood volume are assumed to scale linearly with body mass. Body mass is assumed to scale with age according to the empirical relation derived by Brown et al [3]. The fraction of total organ volume that is cellular is assumed to be 376/485, as assumed by Shah et al [4]. The extracellular volume is assumed to be the difference between the total organ volume and the cellular and vascular volumes. The physiological parameters assumed for the 12-month-old mice are summarised in Supplementary table 3.

Parameter	Value
Total blood volume	1.96 ml
Brain blood flow	0.44 ml/min
Brain blood volume	0.02 ml
Brain organ volume	0.65 ml
Brain cellular volume	0.50 ml
Brain extracellular volume	0.12 ml

Supplementary Table 3: Physiological parameters assumed for the 12-month old mice modelled in Figures 17, 18 and 19 [3, 4].

1.7 Propagation of parameter uncertainty

Uncertainty in parameter estimates were obtained

- from SciPy's curve fit by taking the covariance of the predicted values,
- directly from the output of PINTS' Adaptive Covariance Monte Carlo method.

These were used to obtain estimates of the uncertainty in model outputs by simple bootstrapping: random values of each parameter were drawn from a Gaussian defined by parameter means and standard deviations. These random parameters were used to simulate the experimental time course described in section 1.4. This procedure was repeated 1000 times to gain an estimate of the mean and standard deviation of each numerical output listed in section 1.4, such as the maximum intracellular concentration of CLR01 (Cmax).

1.8 Sensitivity analysis

To better understand how uncertainty in each model parameter impacts upon model outputs (such as the area under the curve), a sensitivity analysis was performed using two techniques: the Fourier Amplitude Sensitivity Test as defined by [5-7] and as implemented in the python package, SALib [8], and a simple local sensitivity analysis.

The local sensitivity analysis was performed by selecting a base set of parameter values, then changing each parameter in turn by 1% or by one standard deviation. The resulting fractional change to each observable listed in section 1.4 due to a change in each parameter was calculated. The Fourier Amplitude Sensitivity Test was run on parameter values ranging from 0.1 times to ten times the same parameter values used for the local sensitivity analysis. The technique yields sensitivity indices and total sensitivity indices for each parameter and observable, which generally yield higher variables for parameters to which an observable is most sensitive. For a given observable, the sensitivity indices would sum to 1.0 if there were no higher-order effects between parameters (that is, variation in the observable that is due to products of parameters). The total sensitivity indices count these higher-order effects for each parameter, so sum to more than 1.0 if there are higher order effects.

2. Fitting, simulation and sensitivity analysis

2.1 Fit to blood kinetics

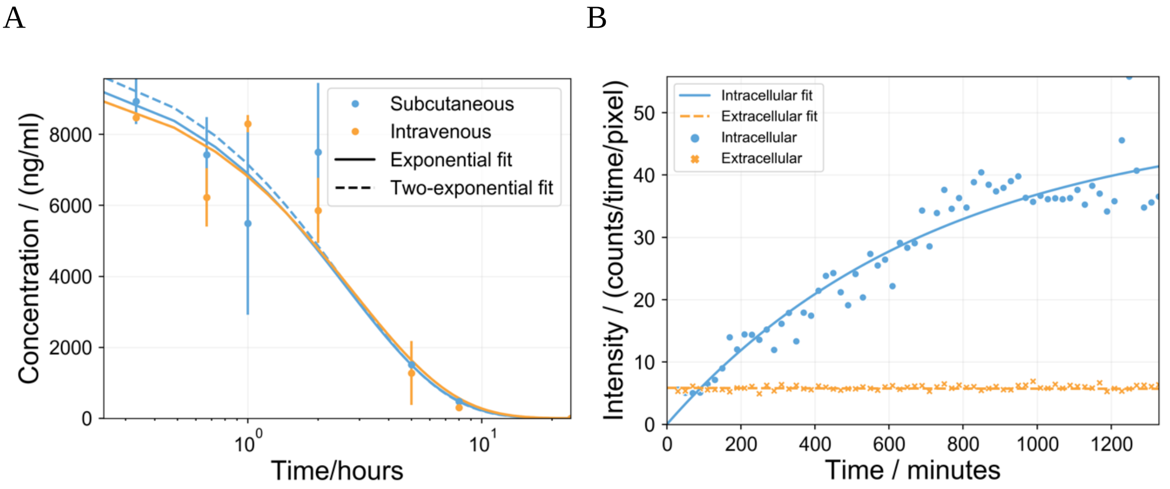

Supplementary Figure 17: A) Fits of an exponential and of two exponentials, Supplementary equations 1 and 2 to clearance kinetics of CLR01 from the blood after subcutaneous or intravenous delivery, n = 3 independent animals. B) Fits of ordinary differential equations, Supplementary equation set 4, to data describing cellular uptake of CLR01 in an in vitro system, n = 1. Data are presented as mean values +/- SD as appropriate.

Supplementary equations 1 and 2 were fit to the concentration of CLR01 in mouse blood after subcutaneous or intravenous delivery, as shown in Figure 17A. The Gaussian, Supplementary equation 3, yielded a poor fit and was discarded. Fits for both sets of data and for both equations are similar and so the exponential fit was selected to describe the loss of CLR01 from the blood, as it requires the fewest parameters. The resulting predicted loss rate from the blood was k = (6.1 ± 1.1) x 10^{-3} / minute, or a half-life of (110 ± 20) minutes.

2.2 Fit to cellular uptake data

A pair of ordinary differential equations, Supplementary equation set 4, were fit to data describing cellular uptake of CLR01 in vitro. The resulting fit is shown in Figure 17B, showing good agreement between the equations and the data. The predicted value for the membrane transfer rate is $\gamma = 8.0 \pm 0.7 \times 10^{-2}$ / hour, or a half-life of 8.6 ± 0.7 hours (in the absence of extracellular CLR01). The predicted value of the equilibrium ratio of intracellular to extracellular concentrations is $\theta = 8.7 \pm 0.4$.

2.3 Fit to pharmacokinetic data in blood and brain

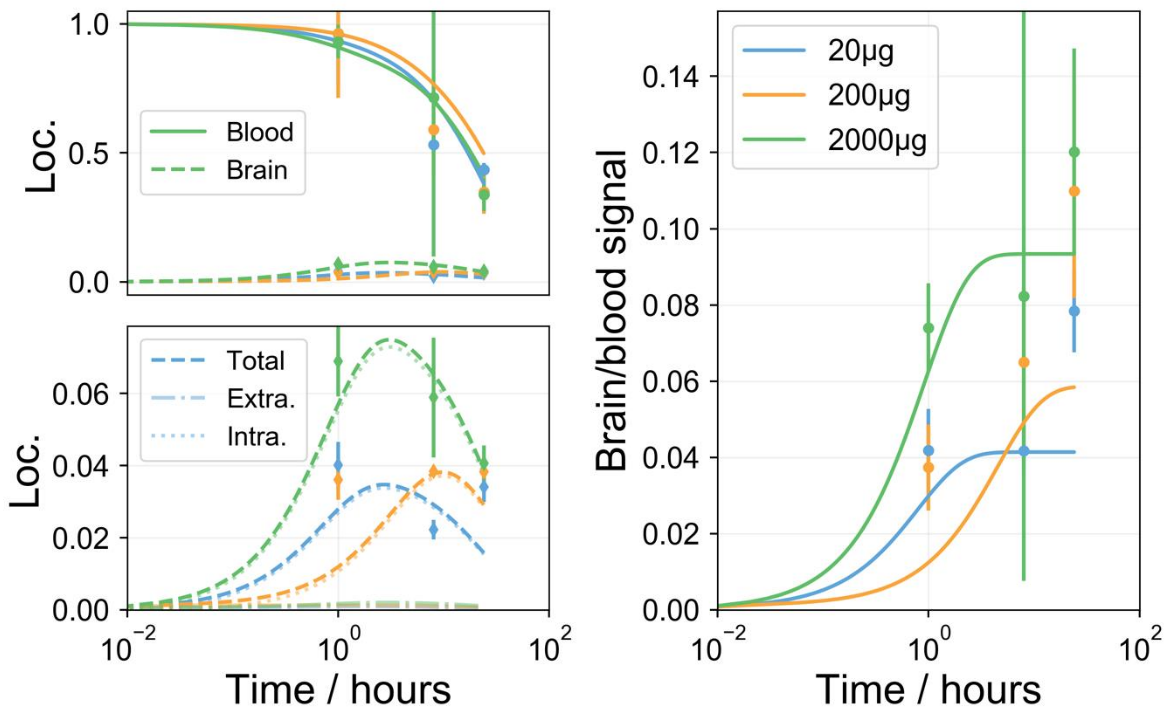

Supplementary Figure 18: Fit of a system of ordinary differential equations, Supplementary equation set 12, to data of the concentration of CLR01 in the blood and brain of 12-month-old mice over time. Scatter points show data and lines show model estimates. The top left panel shows blood and brain relative localisation ("Loc."), such that the initial sum of CLR01 molecules in this plot adds up to 1.0. The bottom left panel shows total, extracellular and intracellular localisation in the brain over same time course. The right panel shows the ratio of brain to blood localisation. The colours have the same meaning for each plot, indicating different initial dosages given to experimental mice, n = 3 independent animals. Data are presented as mean values +/- SD.

The concentration of CLR01 in the blood and brain of 12-month-old mice was measured over the course of 24 hours for three different dosages (Fig. S8A). Supplementary equation set 12 was fit to these data to estimate the rate at which CLR01 crosses the blood brain barrier and the equilibrium ratio of brain/blood concentrations. Examples of such fits are shown in figure 18. Fits to blood data are good but the quality of fit to brain localisation is variable. Fits were especially poor if the rate at which CLR01 crosses the blood-brain barrier is left as a free parameter and the rate at which it crosses cell membranes within the brain is fixed to the value estimated in section 2.2. Instead, the parameter describing entry into the brain, e, was fixed to 0.01, which is the order of magnitude predicted by fits to data. The rate at which CLR01 crosses cell membranes in the brain (γ), the rate of loss of CLR01 from the blood (d) and the equilibrium ratio of brain to blood CLR01 concentrations (μ) were left variable. The equilibrium intracellular/extracellular concentration ratio (θ) was fixed to the value predicted in section 2.2.

Predicted and fixed values are summarised in Supplementary table 4. The parameters μ, d and θ were fit to reasonable accuracy, but the parameter γ is under-determined after averaging over several datasets, due mostly to the very poor fit to the 20µg data. We investigated the likely

consequences of the poor fit of this parameter and of fixing the parameter *e* by performing a sensitivity analysis, as described in section 2.5.

Parameter	Fixed	Value
Blood-brain barrier entry proportion, *e*	Fixed	0.01 ± 0.01
Equilibrium brain/blood concentration ratio, μ	Variable	35.8 ± 5.6
Rate of loss from blood, *d*	Variable	(3.6 ± 1.2) x 10^{-2} / hour
Rate of transfer across cell membranes in brain, γ	Variable	(3 ± 6) x 10^{-1} / hour
Equilibrium intracellular/extracellular concentration ratio, θ	Fixed	8.7 ± 0.4

Supplementary Table 4: The mean and standard deviation of pharmacokinetic parameters estimated from fitting three sets of data described in section 2.3. Parameters that are fixed to specific values or left variable are indicated, as justified in the main text.

2.4 Simulation of dosing regimen

The experimental procedure described in material and methods for the dosing of 12-month-old animals was simulated according to section 1.4, using Supplementary equation set 12 with the parameters defined in Supplementary table 4. The resulting concentration time-course is presented in Figure 19. These results were used to generate observables of interest, such as Cmax, as described in section 1.4. In order to estimate the uncertainty in these observables, 1000 sets of parameter values were generated from Gaussian distributions described by their means and standard deviations, used to generate 1000 time-courses of the experimental procedure, which in turn were used to calculate the mean, standard deviation, median and interquartile range of the observables of interest. These values are displayed in Supplementary table 5. Several observables have a large standard deviation which is not matched by a large interquartile range. This is due to rare extreme values of the parameter *e* from the blood that change the dynamics of the system and cause large predictions of observable values. These rare large values skew the mean and standard deviation. For this reason, the median and IQR are reported with the mean and standard deviation in Supplementary table 5. The dependence of each observable on parameters is explored in more detail in section 2.5.

Observable	Mean ± SD	Mean & IQR	Units
Area under curve (average over brain)	1.4 ± 1.2	1.2 & 0.6	10^5 ngh/ml
Area under curve (intracellular only)	3.2 ± 2.8	2.7 & 1.4	10^5 ngh/ml
Time-averaged area under curve (brain)	100 ± 90	90 & 50	ng/ml

Time-averaged area under curve (intracellular)	240 ± 210	200 & 100	ng/ml
Cmax (average over brain)	230 ± 140	210 & 70	ng/ml
Cmax (intracellular only)	510 ± 310	480 & 160	ng/ml
Half-life of compound in blood*	19 ± 6 -	-	hours
Half-life of compound in brain extracellular space*	0.02 ± 0.04	-	hours
Half-life of compound in brain intracellular space*	6 ± 24	-	hours
Time above intracellular EC50 (averaged across brain)	58 ± 22	54 & 30	% total
Time above intracellular EC50 (intracellular only)	93 ± 11	100 & 12	% total
Time above extracellular EC50 (averaged across brain)	81 ± 17	85 & 31	% total
Time above extracellular EC50 (intracellular only)	98 ± 5	99.9 & 0.2	% total

Supplementary Table 5: Observable quantities calculated from the simulation defined in section 2.4, presented as their mean, standard deviation ("SD"), median and interquartile range ("IQR") over 1000 simulations, using input parameters drawn at random according to their uncertainty. The numerical definition and calculation steps for these values are described in section 1.4. "Total" simulation time in the last four rows is 56.0 days. *Note that half-lives are calculated from parameter values and so the median and IQR are not given. Note also that they are only valid when the compartment described has no input (e.g. an input from the blood to the brain). The actual rate of concentration decrease will be less.

2.5 Sensitivity analysis

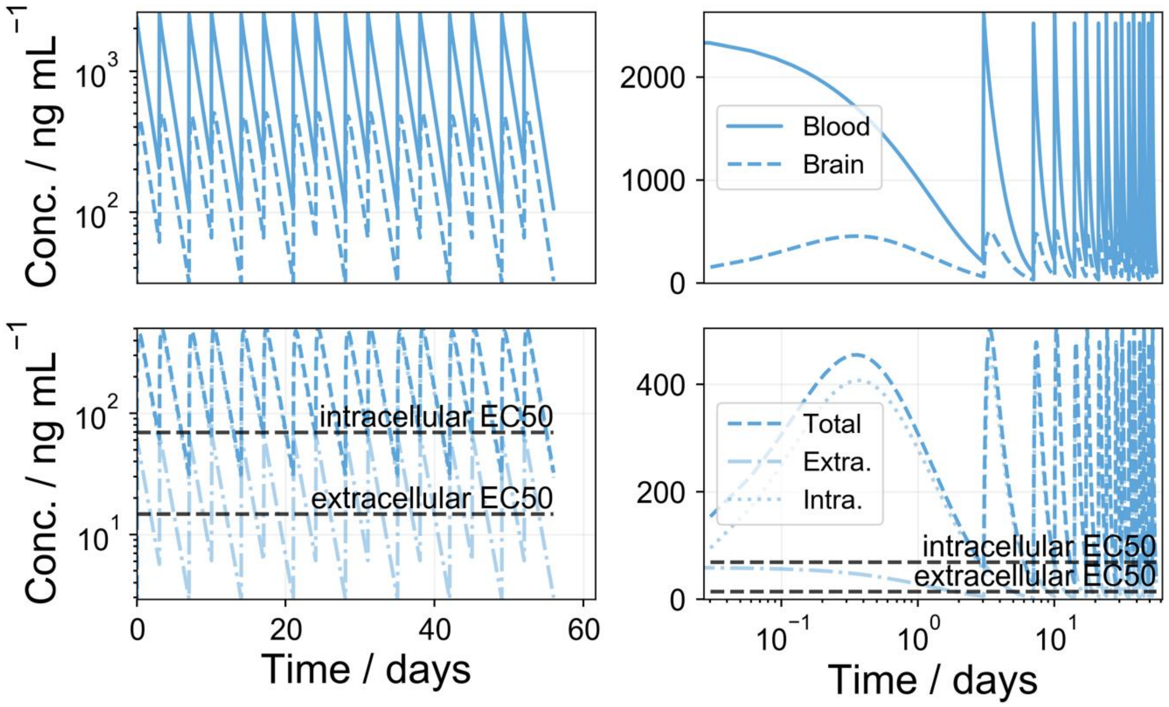

Supplementary Figure 19: A simulation of the time course of the 12-month-old SNCA-OVX cohort, described mathematically in section 1.4, using Supplementary equation set 12 with the parameters defined in Supplementary table 4. Each panel displays the same data, but the top row shows blood and brain concentrations whilst the bottom row shows brain extracellular and intracellular concentrations, and the two columns have different axes logged. The legend for each row is shown on the right-hand side.

To understand how parameter uncertainty affects results, a sensitivity analysis was performed on Supplementary equation set 12 for each observable, except those that depend trivially on input parameters (such as the rate of loss of CLR01 from the blood, which simply equals d). The results, using a Fourier Amplitude Sensitivity Test and two local sensitivity analyses, are shown in Figure 20. Abbreviations used are as follows:

- AUC_b: Area under curve (average over brain)
- AUC_i: Area under curve (intracellular only)
- Cmax_b: Cmax (average over brain)
- Cmax_i: Cmax (intracellular only)
- t>iEC50_b: Time above intracellular EC50 (averaged across brain)
- t>iEC50_i: Time above intracellular EC50 (intracellular only)
- t>eEC50_b: Time above extracellular EC50 (averaged across brain)
- t>eEC50_i: Time above extracellular EC50 (intracellular only).

Results indicate that, before accounting for differing uncertainties, maximum concentrations (Cmax) depend primarily on the equilibrium brain/blood concentration ratio (μ), and other parameters depend primarily on the rate of loss of CLR01 from the blood. A local sensitivity analysis where parameter values are changed by 1% is inconclusive, as each parameter causes similar changes in most cases, or no change in the case of the time that intracellular

concentrations are above the extracellular EC50. When parameters are perturbed by a standard deviation instead of a fixed percentage, the rate of loss of CLR01 from the blood, d, becomes relatively important for all parameters due to its slightly larger uncertainty. Maximum concentrations become equally sensitive to several parameters. Even in this regime, no observable is strongly sensitive to proportion of CLR01 that crosses the blood brain barrier, e, or the rate of transfer across cell membranes in brain, γ, despite their large uncertainties. We hence conclude that the lack of precision in our predicted values for these parameters do not impact our predictions for observable values.

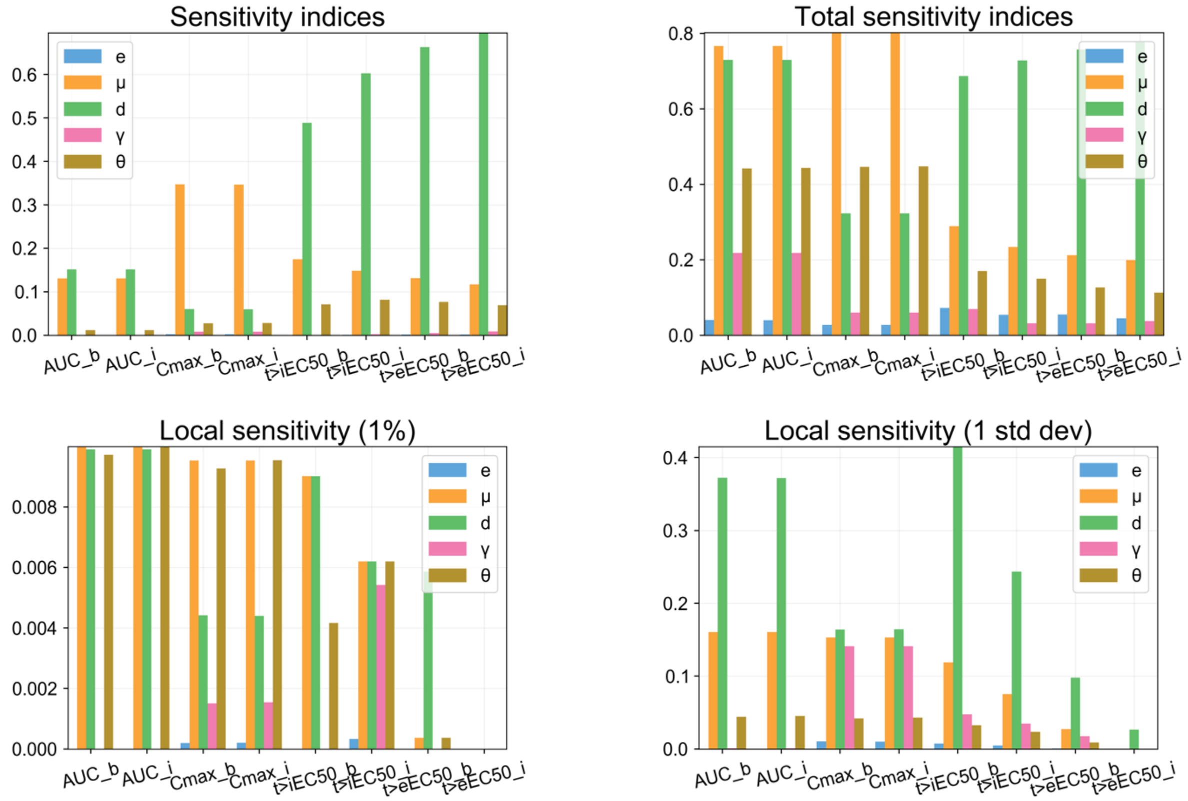

Supplementary Figure 20: Sensitivity analyses of Supplementary equation set 12, as described in section 2.5. Each panel shows a different measure of sensitivity: sensitivity and total sensitivity indices from a Fourier Amplitude Sensitivity Test, and fractional changes after a change in parameter value of 1% or 1 standard deviation. Larger values on the y-axis indicate greater sensitivity. The x-axis shows groups of 5 bars arranged by observables (e.g. "AUC_b" on the left). Bar colours indicate the parameter. Parameter symbols are defined in Supplementary table 3. Abbreviations on the x-axis are listed in the main text.

3. Blood kinetics, cellular uptake and pharmacokinetic analysis

3.1 Blood kinetics

An exponential distribution was selected to describe the loss of CLR01 from the blood (section 2.1). This implies that there is one significant mode of loss for the molecule, though there must be at least two sinks (one to the brain, and the route through which CLR01 is destroyed or lost). It was expected that the sum of two exponentials would provide a better fit than a single

exponential for this reason. The data describing the time-course of CLR01 in both the blood and the brain (section 2.3) had markedly different kinetics; loss of CLR01 from the blood was much slower and the previously estimated value of the loss rate could not fit the data. The parameter had to be re-estimated along with other parameters. Use of the full system of ordinary differential equations (Supplementary equation set 12) removes the need to distinguish between one or two exponentials, but the addition of another free parameter lowers the confidence with which other parameters can be fit.

3.2 Cellular uptake

Parameters relating to uptake of CLR01 by cells within the brain (the equilibrium intracellular/extracellular concentration ratio, θ, and the rate of transfer across membranes, γ) were estimated from fluorescence microscopy data. Relating this data to absolute concentrations of CLR01 is difficult, due to background (noise) levels and the conversion from two-dimensional data to three-dimensional space. Scaling relations for relevant dimensional quantities were calculated (section 1.2.2), to allow simulated 3D concentrations to be related to fluorescence intensities. When comparing extracellular to intracellular fluorescence, background fluorescence is assumed to make up part of the extracellular fluorescence intensity.

3.3 Physiologically-based pharmacokinetics and simulation of experimental time-course

A typical marker for blood brain barrier penetration of a given molecule is its ratio of brain/blood concentrations. At the time points in section 2.3, localisation in the brain is roughly constant and localisation in the blood falls, so the brain/blood signal ratio rises. In the model defined in section 1.3, the ratio of these signals rises at early times before saturating at an equilibrium ratio of concentrations, if the rate of exit from the brain is faster than that from the blood. If the rate of exit from the blood is faster, then this ratio rises exponentially. The best-fit value of the equilibrium intracellular/extracellular concentration ratio, θ, leads to saturation on the timescale of the data, so a poorer fit to some data than if the ratio of signals rose linearly on the timescale of interest. If θ is allowed to take a smaller value, then the fit is improved. However, we could not biologically justify changing the value from that estimated from the fluorescence microscopy data, so instead accepted the poorer fit to the pharmacokinetic data. Including additional biology such as receptor occupancy in the mathematical model may prevent runaway exponential growth of the brain/blood signal ratio, when the rate of loss of CLR01 from the blood is faster than the rate of exit from the brain. A larger amount of data in which the localisation in the brain and the blood are separately measured might also remedy this; most available data only contains the ratio of brain to blood signals. As both of these signals are small, this leads to large relative errors and increases uncertainty in the resulting mathematical fit.

Though curve fit and basinhopping from SciPy [1] and a Bayesian technique from PINTS [2] were all used to fit the data, presented results are the simple curve fit fits, which is the least powerful technique. There are reasons for this. The different time courses lead to significantly different predicted parameter values, which may be because dosage has no effect on dynamics in the model (Supplementary equation set 12). This causes a hierarchical Bayesian fit to yield

parameters that do not strictly fit any of the three datasets and it is not clear, by eye, that predicted parameter values can be trusted. Further work with this model should include biology that leads to dose-dependent CLR01 localisation, such as receptor occupancy. The basinhopping technique was not presented over curve fit as it does not provide as estimate of the covariance of predicted parameter values, complicating the estimation of uncertainty.

Fits to data in which the blood-brain barrier entry parameter, e, was variable did not constrain its value with high confidence. This parameter controls the timescale on which CLR01 enters or leaves the brain's interstitial space from the vasculature, but has no impact on equilibrium concentration in either compartment (as the input rate to the brain is eB/V and the output rate is $e\mu B/\tilde{V}_e$, where B, V and $\tilde{V}_e$ are the brain's blood flow, vascular volume and extracellular volume). The low confidence in the value of e may be because the entry happens over a timescale not captured by the data, or because other effects such as the rate of loss of CLR01 from the blood or the rate of entry into the intracellular compartment dominate it. In particular, measured localisation in the brain does not distinguish between extracellular and intracellular space. Fits to all data gave an order of magnitude for e of 0.01, so e was fixed to this value and the rate of transfer of CLR01 across cellular membranes, γ, was allowed to be variable instead. This improved the confidence in parameter values after fitting to data.

The impact of uncertainty in predicted parameters on observable quantities was measured through the use of bootstrapping (drawing random values of parameters from a Gaussian distribution defined by the mean and standard deviation of predicted parameter values) and sensitivity analyses. These led to the conclusion that the parameters e and γ have little impact on target quantities such as the time for which CLR01 is above the EC50 in the brain. However, the standard deviations assigned to each parameter were estimated from the local covariance of the fit yielded by curve fit, which may not be a measure of the true error (as it does not, for example, quantify uncertainty arising from there being multiple valid solutions for a given parameter value). The error yielded from a bayesian approach could be more reliable, but we opted not to use this approach for the reasons outlined above. Anatomical parameters such as the blood flow to the brain are given fixed values from the literature, with simple scaling relations to account for differences in age. These values may be inaccurate for the mice in this experiment, or may differ between experimental mice. No attempt was made to estimate this additional uncertainty. However, we expect order-of-magnitude agreement between the real and predicted anatomical and pharmacokinetic parameter values, and so we expect order-of-magnitude agreement between real and predicted observable values (such as the area under curve).

Supplementary References

[1] Eric Jones, Travis Oliphant, Pearu Peterson, et al. SciPy: Open source scientific tools for Python, 2001-. URL http://www.scipy.org/. [Online; accessed Apr 2020].

[2] Michael Clerx, Martin Robinson, Ben Lambert, Chon Lok Lei, Sanmitra Ghosh, Gary R Mirams, and David J Gavaghan. Probabilistic inference on noisy time series (PINTS). *Journal of Open Research Software*, 7(1):23, 2019. doi: 10.5334/jors.252.

[3] R P Brown, M D Delp, S L Lindstedt, L R Rhomberg, and R P Beliles. Physiological parameter values for physiologically based pharmacokinetic models. *Toxicology and Industrial Health*, 13(4):407-84, 1997. ISSN 0748-2337. doi: 10.1177/074823379701300401.

[4] Dhaval K. Shah and Alison M. Betts. Towards a platform PBPK model to characterize the plasma and tissue disposition of monoclonal antibodies in preclinical species and human. *Journal of Pharmacokinetics and Pharmacodynamics*, 39(1):67{86, 2012. ISSN 1567567X. doi: 10.1007/s10928-011-9232-2.

[5] A Saltelli, S Tarantola, and K. P.-S. Chan. A Quantitative Model-Independent Method for Global Sensitivity Analysis of Model Output. *Technometrics*, 41(1):39-56, 1999. ISSN 0040-1706. doi: 10.1080/00401706.1999.10485594.

[6] A Saltelli, S Tarantola, and F Campolongo. Sensitivity Analysis as an Ingredient of Modeling. Statistical Science, 15(4):377-395, 2000.

[7] David Makowski, Cedric Naud, Marie-Helene Jeuffroy, Aude Barbottin, and Herve Monod. Global sensitivity analysis for calculating the contribution of genetic parameters to the variance of crop model prediction. *Reliability, Engineering and System Safety*, 91(10-11):1142-1147, 2006. ISSN 09518320. doi: 10.1016/j.ress.2005.11.015.

[8] Will Usher, Jon Herman, Calvin Whealton, David Hadka, xantares, Fernando Rios, bernardoct, Chris Mutel, and Joeri van Engelen. SALib. URL https://doi.org/10.5281/zenodo.160164. [Online; accessed Apr 2020].
