# Supplementary material for: CLR01 protects dopaminergic neurons in vitro and in mouse models of Parkinson’s disease
Source: Nat Commun. 2020 Sep 28;11:4885. doi: 10.1038/s41467-020-18689-x (PMC7522721; doi:10.1038/s41467-020-18689-x)
Supplement: Supplementary file 3 — Reporting summary [file 41467_2020_18689_MOESM3_ESM.pdf]

nature research

Corresponding author(s): Richard Wade-Martins

Last updated by author(s): Aug 14, 2020

# Reporting Summary

Nature Research wishes to improve the reproducibility of the work that we publish. This form provides structure for consistency and transparency in reporting. For further information on Nature Research policies, see our Editorial Policies and the Editorial Policy Checklist.

## Statistics

For all statistical analyses, confirm that the following items are present in the figure legend, table legend, main text, or Methods section.

| n/a | Confirmed | |
|---|---|---|
| ☐ | ☒ | The exact sample size (*n*) for each experimental group/condition, given as a discrete number and unit of measurement |
| ☐ | ☒ | A statement on whether measurements were taken from distinct samples or whether the same sample was measured repeatedly |
| ☐ | ☒ | The statistical test(s) used AND whether they are one- or two-sided<br>*Only common tests should be described solely by name; describe more complex techniques in the Methods section.* |
| ☐ | ☒ | A description of all covariates tested |
| ☐ | ☒ | A description of any assumptions or corrections, such as tests of normality and adjustment for multiple comparisons |
| ☐ | ☒ | A full description of the statistical parameters including central tendency (e.g. means) or other basic estimates (e.g. regression coefficient) AND variation (e.g. standard deviation) or associated estimates of uncertainty (e.g. confidence intervals) |
| ☐ | ☒ | For null hypothesis testing, the test statistic (e.g. *F*, *t*, *r*) with confidence intervals, effect sizes, degrees of freedom and *P* value noted<br>*Give P values as exact values whenever suitable.* |
| ☒ | ☐ | For Bayesian analysis, information on the choice of priors and Markov chain Monte Carlo settings |
| ☒ | ☐ | For hierarchical and complex designs, identification of the appropriate level for tests and full reporting of outcomes |
| ☒ | ☐ | Estimates of effect sizes (e.g. Cohen's *d*, Pearson's *r*), indicating how they were calculated |

*Our web collection on statistics for biologists contains articles on many of the points above.*

## Software and code

Policy information about availability of computer code

| | |
|---|---|
| Data collection | Harmony version 4.9<br>EVOSflauto version 1.7<br>Mercator version 6.5<br>CatwalkXT version 9.1<br>Axoscope version 11.00 |
| Data analysis | ImageJ version 1.52q<br>Axoscope version 11.00<br>Custom code was generated for the evaluation of the pharmacokinetics of CLR01, which is available under request. |

For manuscripts utilizing custom algorithms or software that are central to the research but not yet described in published literature, software must be made available to editors and reviewers. We strongly encourage code deposition in a community repository (e.g. GitHub). See the Nature Research guidelines for submitting code & software for further information.

# Data

Policy information about availability of data

All manuscripts must include a data availability statement. This statement should provide the following information, where applicable:

- Accession codes, unique identifiers, or web links for publicly available datasets
- A list of figures that have associated raw data
- A description of any restrictions on data availability

Authors can confirm that all relevant data are included in the paper and/ or its supplementary information files. The code for the pharmacokinetics modeling in the brain is available upon request. All data is available in the Source Data File.

# Field-specific reporting

Please select the one below that is the best fit for your research. If you are not sure, read the appropriate sections before making your selection.

☒ Life sciences ☐ Behavioural & social sciences ☐ Ecological, evolutionary & environmental sciences

For a reference copy of the document with all sections, see nature.com/documents/nr-reporting-summary-flat.pdf

# Life sciences study design

All studies must disclose on these points even when the disclosure is negative.

**Sample size**

Sample size was determined according to the experimental paradigm.
- 16 animals were bread in order to account for loss of animals during ageing and avoid n < 10 at the end of the study where behavioral differences of ageing cohorts were investigated. Any animals showing any welfare issues were taken off the study, otherwise animals remained in the sample.
- For neuropathological examination 4-5 animals were bred per condition in order to avoid n < 3.
- For iPSC culture, 3-4 independent control lines were used in order to ensure n > 3.
- For experiments using cell lines or primary cultures 3 independent replicates were used in order to allow statistical analysis.
- For biochemical analysis of recombinant or biological material 3 independent experiments were performed on 3 independent samples.
- For fast-cyclic voltammetry 5 animals were used per condition.

These numbers have been determined by our experience from previous reports including, Janezic et al. 2013, Sloan et al. 2016, Attar et al. 2014, Roberts et al. 2015., and Lang et al. 2018.

**Data exclusions**

Any animals showing any welfare issues were taken off the study, otherwise animals remained in the sample. Exclusion criteria were pre-established and include:

- Any welfare issues including pilorection, grimace, abnormal spontaneous activity, discharge or other signs of infection, etc. Figures S11A-D, S13A-D,M

- For analyses involving gait: broken fingers, toes or nails and impaired vision (due to conjunctivitis or missing, cloudy, or receding eyes). Figure 4A.

Any tissue which was damaged or did not meet quality control for the appropriate region/s for analysis was discarded. When insufficient tissue was available from an animals, experiments were run on all remaining animals. Figures 3B/F, 4F/H, 6F/H, S14B/C.

Any outliers were determined by Grubb's test, otherwise all data points were maintained in the study. Figure 4B.

Any wells of cells showing overt signs of bacterial or fungal contamination were removed from analysis. Figure S6C.

**Replication**

Experiments involving animals were performed once with the indicated number of independent animals. Experiments involving cell lines were replicated three times in order to be able to perform statistical analysis. Replication of these experiments was successful.

**Randomization**

Animals were randomly assigned to treatment once, sex, weight and aged matched.

For cell-based experiments randomization does not apply as all available lines were treated in the same fashion.

**Blinding**

All non-automated analysis was blinded to the researcher, by taping over or removing sample identifiers, as appropriate. All blinding was performed before data collection, and samples were unblinded post-analysis.

# Reporting for specific materials, systems and methods

We require information from authors about some types of materials, experimental systems and methods used in many studies. Here, indicate whether each material, system or method listed is relevant to your study. If you are not sure if a list item applies to your research, read the appropriate section before selecting a response.

## Materials & experimental systems

| n/a | Involved in the study |
|---|---|
| ☐ | ☒ Antibodies |
| ☐ | ☒ Eukaryotic cell lines |
| ☒ | ☐ Palaeontology and archaeology |
| ☐ | ☒ Animals and other organisms |
| ☐ | ☒ Human research participants |
| ☒ | ☐ Clinical data |
| ☒ | ☐ Dual use research of concern |

## Methods

| n/a | Involved in the study |
|---|---|
| ☒ | ☐ ChIP-seq |
| ☒ | ☐ Flow cytometry |
| ☒ | ☐ MRI-based neuroimaging |

## Antibodies

Antibodies used

As these antibodies are routinely used in the lab, the lot numbers used for this study are unavailable as we have gone through the whole aliquots.

Mouse monoclonal anti-alpha-syn4D6, ab1903, Abcam, 1:2000
Mouse monoclonal anti-dynein MAB1618, Millipore, 1:100
Mouse monoclonal anti-kinesin MAB1614, Millipore, 1:100
Rabbit anti-GFAP Z0334, Sigma, 1:1000
Rabbit anti-Iba1 019-19741,Wako, 1:1000
Rabbit anti-TH ab152, Millipore, 1:1000
Chicken anti-TH ab76442, Abcam, 1:100
Chicken anti-Tuj1 ab107216, Abcam, 1:100
Rabbit anti-phospho-alpha-synuclein EP1532Y, Abcam, 1:1000
Mouse anti-alpha-syn SYN1 clone 42 610787, BD Transduction Laboratories, 1:1000
Mouse anti-aggregated alpha-syn antibody Syn-F1, BioLegend, 1:2000
Mouse human α-syn-specific antibody clone syn211, 32-8100, Thermo Scientific, 1:1000
Goat anti-mouse TRITC-conjugated antibody, 115-025-146, Jackson, 1:500

All secondary Alexa antibodies were used at 1:1000 and acquired from Thermo Scientific

Validation

Mouse monoclonal anti-!-syn4D6, ab1903, Abcam --> Validated on human and mouse tissue for appropriate staining pattern in PD brain or PD mouse model
Mouse monoclonal anti-dynein MAB1618, Millipore --> Validated in vitro reproducing appropriate staining pattern
The antibody was serially diluted in order to ensure a dose dependent staining pattern that matched the staining pattern shown by the provider and that of the publications shown on the providers website. Once that was established, the antibody was conjugated and a serial dilution of it as well as one for alpha-synuclein, were tested in order to detect the best combination of antibodies. No ligase, plus and minus probes were run in parallel to ensure no unspecific proximity ligation. Finally, the assay was run in cells expressing endogenous alpha-synuclein and cells overexpressing alpha-synuclein in order to further address specificity.
Mouse monoclonal anti-kinesin MAB1614, Millipore --> Validated in vitro reproducing appropriate staining pattern
The antibody was serially diluted in order to ensure a dose dependent staining pattern that matched the staining pattern shown by the provider and that of the publications shown on the providers website. Once that was established, the antibody was conjugated and a serial dilution of it as well as one for alpha-synuclein, were tested in order to detect the best combination of antibodies. No ligase, plus and minus probes were run in parallel to ensure no unspecific proximity ligation. Finally, the assay was run in cells expressing endogenous alpha-synuclein and cells overexpressing alpha-synuclein in order to further address specificity.
Rabbit anti-GFAP Z0334, Sigma --> Validated in tissue (mouse and human) reproducing appropriate staining pattern
Rabbit anti-Iba1 019-19741,Wako --> Validated in tissue (mouse and human) reproducing appropriate staining pattern
Rabbit anti-TH ab152, Millipore --> Validated in tissue (mouse and human) reproducing appropriate staining pattern
Chicken anti-TH ab76442, Abcam --> Validated in tissue (mouse and human) reproducing appropriate staining pattern
Chicken anti-Tuj1 ab107216, Abcam --> Validated in tissue (mouse and human) reproducing appropriate staining pattern
Rabbit anti-phospho-alpha-synuclein EP1532Y, Abcam --> Validated on human and mouse tissue for appropriate staining pattern in PD brain or PD mouse model
Mouse anti-alpha-syn SYN1 clone 42 610787, BD Transduction Laboratories --> Validated on human and mouse tissue for appropriate staining pattern in PD brain or PD mouse model
Mouse anti-aggregated alpha-syn antibody Syn-F1, BioLegend --> Validated on human and mouse tissue for appropriate staining pattern in PD brain or PD mouse model
Mouse human α-syn-specific antibody clone syn211, 32-8100, Thermo Scientific --> Validated on human and mouse tissue for appropriate staining pattern in PD brain or PD mouse model

# Eukaryotic cell lines

Policy information about cell lines

| | |
|---|---|
| Cell line source(s) | ATCC SH-S5Y5 |
| Authentication | SH cells were karyotyped and STR profiled by the supplier. We did not re-authenticate in house. |
| Mycoplasma contamination | Cell lines tested negative for micoplasma. |
| Commonly misidentified lines (See ICLAC register) | No commonly misidentified cell lines were used in the study. |

# Animals and other organisms

Policy information about studies involving animals; ARRIVE guidelines recommended for reporting animal research

| | |
|---|---|
| Laboratory animals | All mouse groups were age, sex (both genders) and weight matched. All animals were either C57/Bl6 or alpha-synuclein overexpressing transgenic animals based on the same background. Animals were 3, 6, 9, 12 or 18 month old (+/- 2 months from 12 months of age) as specified for each experiment. |
| Wild animals | No wild animals were used in this study |
| Field-collected samples | No field collected samples were used in this study. |
| Ethics oversight | All procedures were conducted in accordance with the United Kingdom Animals (Scientific Procedures) Act of 1986 and approved by the local ethical review panel at the Department of Physiology, Anatomy and Genetics, University of Oxford, or in accordance with in accordance with the European Union directive of September 22, 2010 (2010/63/EU) on the protection of animals used for scientific purposes, with approval from the Institutional Animal Care and Ethical Committee of Bordeaux University (CE50, France) under the license number 5012099-A. |

Note that full information on the approval of the study protocol must also be provided in the manuscript.

# Human research participants

Policy information about studies involving human research participants

| | |
|---|---|
| Population characteristics | Human substantia nigra pars compacta (SNpc) was dissected from fresh frozen post-mortem midbrain samples from five male patients with sporadic Parkinson's disease exhibiting conspicuous nigral Lewy body pathology on neuropathological examination. Mean age at death: 75 ± 2.75 years; frozen post-mortem interval: 31.8 ± 7.45 h; GIE Neuro-CEB BB-0033-00011. |
| Recruitment | The samples were obtained from the Brain Bank GIE NeuroCEB (BRIF number 0033-00011), funded by the patients' associations France Alzheimer, France Parkinson, ARSEP, and 'Connaître les Syndromes Cérébelleux' to which we express our gratitude. The consents were signed by the patients themselves or their next of kin in their name, in accordance with the French Bioethical Laws. The Brain Bank GIE NeuroCEB (Bioresource Research Impact Factor number BB-0033-00011) has been declared at the Ministry of Higher Education and Research and has received approval to distribute samples (agreement AC-2013-1887). |
| Ethics oversight | The human brain samples were obtained from brains collected in a Brain Donation Program of the Brain Bank "GIE NeuroCEB" run by a consortium of Patients Associations: ARSEP (association for research on multiple sclerosis), CSC (cerebellar ataxias), France Alzheimer and France Parkinson. The consents were signed by the patients themselves or their next of kin in their name, in accordance with the French Bioethical Laws. The Brain Bank GIE NeuroCEB (Bioresource Research Impact Factor number BB-0033-00011) has been declared at the Ministry of Higher Education and Research and has received approval to distribute samples (agreement AC-2013-1887). |

Note that full information on the approval of the study protocol must also be provided in the manuscript.
